# Autonomic Changes During an Adaptive Cognitive Task

**DOI:** 10.3390/jcm15208068

**Published:** 2026-10-17

**Authors:** Maciej Lachowicz, Iwona Wilk, Igor Hrehorowicz, Szymon Bałamucki, Michał Lisowski, Grzegorz Żurek, Waldemar Andrzejewski

**Affiliations:** 1 Institute of Health Studies, DSW Ideis University, 53-611 Wroclaw, Poland; 2 Department of Fundamentals of Physical Therapy and Occupational Therapy, Wroclaw University of Health and Sport Sciences, 51-612 Wroclaw, Poland; 3 Department of Biological Principles of Physical Activity, Wroclaw University of Health and Sport Sciences, 51-612 Wroclaw, Poland

**Keywords:** autonomic reactivity, heart rate, electrodermal activity, cognitive testing, response accuracy, cognitive performance

## Abstract

**Background/Objectives:** This study examined between-participant associations of task-minus-baseline heart rate (HR) and skin conductance (SC) changes with performance during the adaptive Determination Test (DT), in which performance influences subsequent task demand. **Methods:** This exploratory secondary analysis included 48 young adults. Accuracy was the study-defined proportion correct/(correct + incorrect + omitted), calculated from exported counts. Ten Pearson correlations formed the primary analytical family. Complementary fractional-logit and linear models included the corresponding baseline physiological value and the exported stimulus count (DT_Stimuli). Tonic skin conductance level and skin conductance response (SCR) frequency and amplitude were also examined. Benjamini–Hochberg (BH) adjustment was applied separately to the defined analytical families. **Results:** Mean HR increased by 19.99 bpm and mean SC by 4.65 µS. The HR–accuracy Pearson estimate was r = 0.338 (95% CI 0.060 to 0.568; *p* = 0.019; BH q = 0.187), compared with Spearman ρ = 0.192. In the model including baseline HR and DT_Stimuli, the average marginal association was 1.42 percentage points per 10 bpm (95% bootstrap CI 0.13 to 2.65; *p* = 0.033; BH q = 0.306). Neither the primary correlation family nor the 15 complementary model tests met q < 0.05. Direct comparisons did not establish different HR and SC correlations. **Conclusions**: HR and SC increased during adaptive DT performance. A possible HR–accuracy association depended on analytical specification. Adjustment for aggregate stimulus count did not identify the direction of the performance–demand–physiology relationship. These findings support prospective testing with time-resolved task exposure.

## 1. Introduction

Maintaining cognitive performance under time pressure requires sustained attention and the timely selection of appropriate actions. As demands increase, additional effort can sustain performance at a higher physiological cost [1]. Response strategies may also shift toward decisions based on less accumulated information, trading accuracy for speed [2,3]. Accuracy and reaction time therefore describe the outcome of cognitive processing without fully explaining how that outcome was maintained. Examining physiological activity alongside behavior can help characterize the adjustment from rest to active task engagement and whether that adjustment accompanies more effective performance.

This physiological adjustment is part of the broader regulation of goal-directed behavior. Cognitive control organizes attention and action in relation to current goals, while autonomic regulation adjusts bodily state to the demands of engagement. These processes involve partly overlapping brain systems, including the anterior cingulate and insular cortices [4]. In a study combining brain imaging with cardiac recording, Critchley et al. linked anterior cingulate activity to cardiovascular regulation during cognitive and motor tasks. Patients with damage involving this region showed blunted cardiovascular responses to mental stress despite broadly preserved performance on neuropsychological tests [5]. This convergence provides a biological rationale for studying cognition and physiology together, while also showing that their observable outputs can dissociate. Physiological recruitment may accompany the effort used to sustain performance, the difficulty encountered, or preparation for action. Reviews of workload research accordingly find that sensitivity varies across tasks and physiological signals [6,7]. A behavioral score is therefore more informative when considered alongside the demands under which it was achieved and the physiological response accompanying it.

Cardiovascular responses provide one route to studying this adjustment, but their interpretation depends on the task and the physiological endpoint. A meta-analysis of cardiac measures found that sensitivity to cognitive workload varied across measures and task conditions [8]. Theories of effort mobilization place these responses in the context of task difficulty and the value of successful performance, with cardiac contractility providing much of the supporting evidence [9]. Physiological recruitment and behavioral success therefore need to be examined together. This motivates a specific question: when participants face a demanding cognitive task, does a larger physiological adjustment accompany more effective responding or primarily indicate the demands encountered while responding?

Heart rate (HR) and skin conductance (SC) provide complementary ways to examine this question. HR summarizes cardiac activity influenced by both sympathetic and parasympathetic regulation [10]. SC reflects sympathetic control of sweat gland activity [11,12]. Recording both permits comparison of cardiac and electrodermal changes during the same cognitive challenge without assuming that they carry identical information. For electrodermal activity (EDA), mean SC describes the overall conductance level, while separating its tonic and phasic components distinguishes sustained changes from brief responses [13].

Two hypothetical examples illustrate why physiological activity should be interpreted alongside performance. In a fixed-pace memory task, two participants could achieve similar accuracy while showing different physiological responses. An exploratory study identified high and average-to-high accuracy profiles with different ECG-derived workload estimates; these estimates combined HR and heart rate variability (HRV) rather than measuring HR change alone [14]. Such findings motivate joint assessment without establishing which participant has greater cognitive ability. In a rapid choice response task, errors might coincide with brief electrodermal responses and transient cardiac slowing. This pattern has been observed experimentally [15] and shows why greater electrodermal activity need not indicate better performance. A small average HR change would likewise remain ambiguous without behavioral and temporal context. These examples illustrate the value of evaluating physiological activity against the achieved result, the demands experienced, and the timing of performance events.

The adaptive Determination Test (DT) offers a setting in which cognitive demands change with the participant’s own responses. Participants must identify visual and auditory signals and select the corresponding manual or pedal response while the presentation tempo adjusts to their recent performance [16]. Faster correct responding can produce faster subsequent presentation, whereas errors and missed responses can slow it. Maintaining performance thus requires continuing adjustment to a task whose demands partly arise from earlier performance. A participant who responds effectively may experience more stimulation and make more movements, creating a reciprocal relationship among performance, task demand, and physiological activity.

This feature distinguishes the DT from studies that impose the same predefined difficulty levels on each participant. Such experiments show that physiology can discriminate levels of cognitive demand [17], but they do not establish that individuals with larger physiological changes will perform better. Responses to manipulated demand and differences between individuals answer different questions [18,19]. The latter question is relevant to the use of physiology alongside cognitive assessment, including its proposed application in populations with cognitive impairment [20]. Before clinical interpretation is possible, the relationship between physiological change and behavior needs to be characterized in the context of the task that generates both.

The present exploratory study examined how task-minus-baseline HR and SC changes were associated with cognitive performance during the adaptive DT in healthy young adults. Accuracy was considered alongside response counts and reaction time to distinguish effective responding from speed and response volume. Tonic and phasic EDA measures were examined to determine whether separating electrodermal components clarified their relationship with performance. Analyses accounting for baseline physiology and aggregate stimulus exposure, together with direct comparisons of HR and SC associations, addressed whether the observed pattern was consistent across these complementary perspectives.

## 2. Materials and Methods

### 2.1. Participants and Study Design

The study examined the seated baseline and adaptive DT phases of a laboratory dataset collected in April–May 2026. Of 52 recruited young adults, two did not meet eligibility criteria, and two were excluded following complete physiological recording failures that left no recoverable data for phase-level analysis. The analytical cohort (Table 1) comprised 48 participants (28 women and 20 men), aged 18–23 years (M = 19.60, SD = 1.11).

Eligibility was established through questionnaire screening for adulthood, self-reported good general health, and absence of contraindications. Screening addressed neurological and psychiatric history, other conditions relevant to autonomic measurement, and medications or substances affecting the central or autonomic nervous system. All included participants reported no relevant medication use and adherence to the required abstinence from caffeine and nicotine for 4 h and intensive exercise for 12 h before testing. The questionnaires also recorded sleep duration and sleepiness and were complete for the analytical cohort.

The study adhered to the ethical standards outlined in the Helsinki Declaration of 1964 and its subsequent revisions. Approval for the study’s methods and protocol (approval no. 46/2025, signed 28 January 2026; available in Supplementary File S1) was obtained from the Research Ethics Committee of the Wroclaw University of Health and Sport Sciences. All participants provided written informed consent before participation.

For *n* = 48, the Fisher-z standard error for a correlation is 1/√ (*n* − 3) = 0.149; a correlation near zero therefore has an approximate 95% interval of ± 0.28. Using a two-sided α = 0.05 sensitivity calculation, 80% power corresponds to |r| ≈ 0.39. These values characterize the precision and sensitivity of the sample. Reporting followed STROBE recommendations [21].

### 2.2. Physiological Recording and Processing

Recordings used ConsensysPRO v2.0.23 and Shimmer3 GSR (Shimmer Research Ltd., Dublin, Ireland). EDA and raw photoplethysmography (PPG) were sampled at 128 Hz. Original Shimmer SC electrodes (Shimmer Research Ltd., Dublin, Ireland) were placed on the proximal phalanges of the index and middle fingers of the non-dominant hand. The optical pulse sensor was on the left earlobe. HR was derived using the manufacturer’s PPG-to-HR algorithm. PPG measures peripheral blood volume changes during the cardiac cycle [22].

Procedural event markers identified the seated baseline and DT phases; instruction and transition intervals were excluded. Phase means were arithmetic averages of retained HR and SC samples. SC samples were excluded when missing, outside the sensor range of 0.2–125 µS, or accompanied by movement-related discontinuities in synchronized inertial channels. Negative HR placeholders and values above 200 bpm were excluded without interpolation.

For the present analysis, HR completeness was checked in the exported interval summaries. A complete record required at least 290 s of baseline and 400 s of task recording, with a finite mean HR in every exported interval. The duration thresholds were obtained by rounding the observed minimum phase lengths down to the nearest 10 s. Coverage was defined as the proportion of exported intervals with an available HR mean; it describes summary availability rather than retention of individual PPG samples.

For EDA decomposition, non-finite values and SC values outside 0.2–125 µS were excluded. Abrupt adjacent-sample changes greater than 0.25 µS were marked with a 1 s margin on each side. Constant-value runs lasting at least 10 s were also excluded. Gaps exceeding 1.5 times the median sampling interval defined segment boundaries. Segments lasting at least 30 s were low-pass filtered at 2 Hz using a fourth-order Butterworth filter applied in both directions and resampled to 8 Hz without interpolation across rejected gaps. Tonic and phasic components were estimated with the cvxEDA model [23]. The sparse primal–dual solver implemented in SciPy 1.17.0 used time constants of 2.0 and 0.7 s, 10 s tonic spline knots, α = 0.0008, and γ = 0.01. Segments were standardized for fitting and returned to µS.

Tonic skin conductance level (SCL) was defined as the mean of retained tonic samples. Skin conductance response (SCR) amplitude was the difference between a local phasic maximum and its preceding minimum within the same retained segment. A threshold of 0.05 µS was used for SCR detection. SCR frequency was response count per analyzable minute; mean SCR amplitude was averaged across detected responses. All analyzed phases contained detected responses. Reported phase eligibility required confirmed boundaries, non-negative retained tonic estimates, and at least 80% retained coverage after boundary margins, following a study-specific quality assessment approach [24]. For the present reanalysis, exported analyzable duration was checked retrospectively against phase-specific completeness thresholds of 280 s at baseline and 390 s during the DT. In retained exports, the duration fraction was analyzable duration divided by full phase duration, distinct from raw sample retention.

Change scores were defined as task minus baseline. ΔHR was expressed in bpm; ΔSC and ΔSCL in µS; ΔSCR frequency in responses/min; and ΔSCR amplitude in µS. These variables represent task-related physiological change without assigning a single underlying process.

### 2.3. Procedure and Behavioral Outcomes

After sensor placement and seated stabilization, participants completed a 5 min seated resting baseline, standardized instructions, and the approximately 8 min S2 adaptive long form of the DT. Laboratory sessions were scheduled between 10:00 and 15:00 in the same room, with an ambient temperature of approximately 24 °C.

Participants responded as quickly and accurately as possible to visual and auditory stimuli using the Vienna Test System (VTS 8.19.01; SCHUHFRIED GmbH, Mödling, Austria) response panel and foot pedals. The non-dominant hand bearing SC electrodes did not operate the panel. Visual stimuli appeared on a 17.3 inch LCD screen (1920 × 1200 pixels) approximately 45 cm away. Auditory stimuli were delivered through speakers. Adaptive and scoring definitions follow the DT manual [25]. In adaptive S2 mode, stimulus duration is calculated from the mean of the preceding eight reaction times. If a response is not correct, twice that stimulus’s presentation duration is substituted for its registered reaction time when calculating the next stimulus duration.

The exported stimulus count (DT_Stimuli) is the number of stimuli presented during the test. The response category total (DT_Total) was calculated as correct + incorrect + omitted. These counts differ: the scoring rules allow more than one incorrect reaction to a stimulus and a correct reaction alongside incorrect reactions, while omissions record missed stimuli. DT_Total therefore sums scoring categories rather than distinct stimulus trials or executed motor responses. The presented-stimulus count was used to account for aggregate exposure in adjusted models. An alternative HR–accuracy model used DT_Total in its place to assess sensitivity to the count used for adjustment.

Accuracy was calculated as DT_Correct/DT_Total for all analyses. This study-defined proportion includes omissions in the denominator and differs from the manufacturer’s primary S2 score, the correct response count. Accuracy and the analogous omission and incorrect response proportions were stored on a 0–1 scale; descriptive accuracy is shown as a percentage.

Median reaction time was the participant-level VTS median of all correct reactions, including on-time and delayed reactions, measured from stimulus appearance to a response panel button press and converted from seconds to milliseconds. The exported median was analyzed without additional trial-level latency exclusions. Correct response count was the raw score labelled reactive stress tolerance in the VTS report and includes on-time and delayed correct reactions.

## 3. Statistical Analysis

Inference emphasized effect estimates and confidence intervals. The primary ten-test family and each defined sensitivity family were evaluated with separate Benjamini–Hochberg (BH) adjustments. The families were defined for this exploratory reanalysis according to analytical purpose: unadjusted associations, associations conditional on baseline and exposure, sensitivity to model specification, and direct HR–SC comparisons. Separate adjustments support interpretation within these questions; they do not provide a single study-wide multiplicity correction.

The primary analytical family comprised ten Pearson correlations: two physiological changes (HR and mean SC) by five DT outcomes. Pearson estimates were accompanied by Fisher-z 95% confidence intervals and two-sided tests with 46 degrees of freedom [26]. Benjamini–Hochberg adjustment was applied across the ten tests, with q < 0.05 as the multiplicity threshold [27]. Effect estimates and confidence intervals were interpreted alongside p values [28]. Ten baseline-adjusted ordinary least-squares (OLS) models, each containing physiological change and its corresponding baseline level, were treated as sensitivity analyses. Their change coefficients received a separate BH correction, with type 3 heteroskedasticity-consistent (HC3) covariance estimates used for t-based inference [29].

Complementary models included physiological change, the corresponding baseline level, and DT_Stimuli/100. Fractional-logit quasi-likelihood with a logit link and equal participant weights was used for accuracy, omission proportion, and incorrect response proportion [30]. Median reaction time was analyzed with OLS. Eight models evaluated HR and mean SC across these four outcomes. Six further models evaluated SCL, SCR frequency, and SCR amplitude for accuracy and omission proportion. One interaction model included the product of mean-centered HR change/10 and baseline HR/10, together with both main effects and DT_Stimuli/100; both cardiac predictors were therefore expressed per 10 bpm. The 15 focal coefficients constituted a separate BH family.

HC3 standard errors and *t* tests used *n* minus the number of model parameters as degrees of freedom: 44 for additive models and 43 for the interaction. For proportional outcomes, average marginal derivatives were expressed in percentage points per 10 bpm for HR, per 1 µS for SC and SCL, per 1 SCR/min for SCR frequency, and per 0.1 µS for mean SCR amplitude. The amplitude model was fitted per 1 µS and rescaled for presentation. Median reaction time slopes were expressed in milliseconds, and the interaction was reported on the logit scale. Model effect 95% percentile intervals used 4999 paired participant bootstrap resamples. The bootstrap intervals and HC3 p values represent distinct inferential summaries; family-level interpretation was based on the corresponding q values.

Sensitivity analyses evaluated distributional form, influence, and exposure specification. Spearman correlations received a separate ten-test BH correction. Theil–Sen slopes were accompanied by 95% percentile intervals from 1999 paired participant bootstrap resamples [31]. Leave-one-participant-out analyses were accompanied by residual, influence, and variance inflation diagnostics [32]. The HR–accuracy model was repeated after adding sex with either KSS or sleep duration. Negative binomial models for omission and incorrect response counts included physiological change and corresponding baseline physiology, with log (DT_Stimuli) as an offset. Incidence rate ratios and HC3 intervals were interpreted descriptively. Supplementary Tables S1–S6 report the influence analyses, alternative exposure specifications, and related diagnostics.

Corresponding HR and SC correlations were compared with the Williams test for dependent correlations [33]. Five comparisons formed a separate BH family. Correlation differences had 95% bias-corrected and accelerated (BCa) intervals from 19,999 paired participant resamples [34]. Mean within-participant physiological changes had t-based 95% intervals. All analyses were two-sided and used R 4.4.1 with sandwich 3.1-0, MASS 7.3-60.2, boot 1.3-30, and jmv, with results organized in jamovi 2.6.44. Bootstrap sampling used seed 4,596,147, reset before each model or comparison, with participants ordered by Subject_ID. Percentile intervals used R type 7 quantiles, and BCa intervals used boot::boot.ci. Supplementary Tables S1–S6 distinguish the primary and complementary analytical families.

During preparation of this work, the authors used ChatGPT 5.6 (OpenAI) for language editing and editorial refinement. The authors reviewed and edited all AI-assisted output and take responsibility for the analyses and final manuscript.

## 4. Results

### 4.1. Descriptive Statistics and Signal Quality

**Figure 1:**
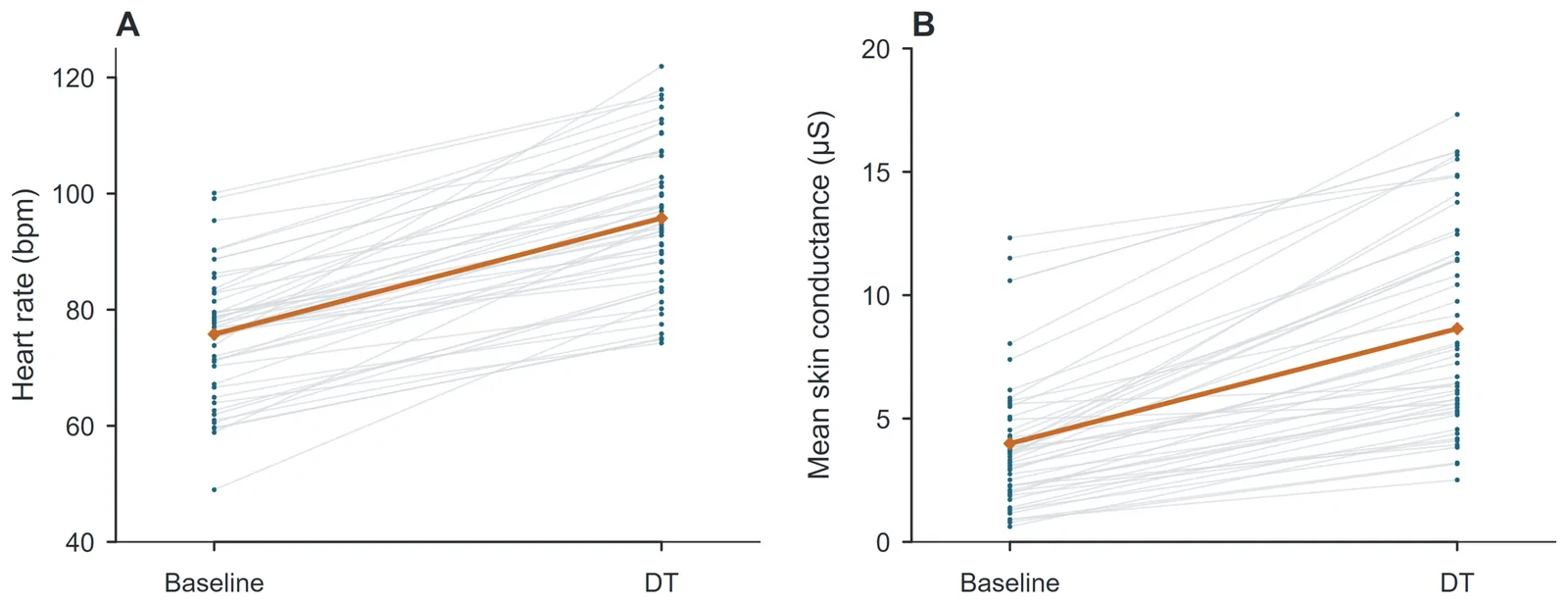
HR and mean SC at baseline and during the DT. Panels (**A**,**B**) show HR and mean SC, respectively. Gray lines connect the two measurements from each participant (*n* = 48); blue points show individual observations and orange diamonds show cohort means. Both task-minus-baseline changes were positive in every participant.

All reported main analyses included 48 participants. Mean HR increased from 75.78 to 95.77 bpm (mean change 19.99; 95% CI 17.63 to 22.35) and mean SC from 3.99 to 8.64 µS (mean change 4.65; 95% CI 3.92 to 5.39). Both changes were positive in every participant (Table 2; Figure 1). Mean tonic SCL increased by 4.37 µS, SCR frequency by 6.60 responses/min, and mean SCR amplitude by 0.055 µS (Table 2). Behavioral performance and stimulus exposure are summarized in Table 3.

Mean accuracy was 90.81% (SD 3.08), and the mean of participant median reaction times was 734.62 ms (SD 54.32). DT_Stimuli ranged from 474 to 650, whereas DT_Total ranged from 483 to 689. DT_Total exceeded DT_Stimuli in all participants by 2–76; accordingly, DT_Stimuli was used as the exposure index. The correlation of HR change with DT_Stimuli was r = 0.083 (95% CI −0.206 to 0.359), and the correlation with correct plus incorrect response counts was r = 0.102 (95% CI −0.188 to 0.375). Processed EDA summaries were available for all 96 baseline and task phases. Exported analyzable EDA duration was 295.44 ± 5.67 s at baseline and 487.24 ± 15.01 s during the DT (mean ± SD). The exported analyzable duration fraction, calculated as analyzable duration divided by the full exported phase duration, was 96.65–100% at baseline and 95.47–100% during DT. All 96 fractions exceeded 95%. These fractions describe exported duration, not raw sample retention.

All 48 participants met the HR completeness criteria. Mean HR was available in all 717 exported intervals (287 baseline and 430 task intervals), giving 100% interval summary coverage in each of the 96 phases. The recorded physiological phases lasted 304.10 ± 5.89 s at baseline and 497.94 ± 14.42 s during the DT (mean ± SD).

### 4.2. Primary Correlations

**Figure 2:**
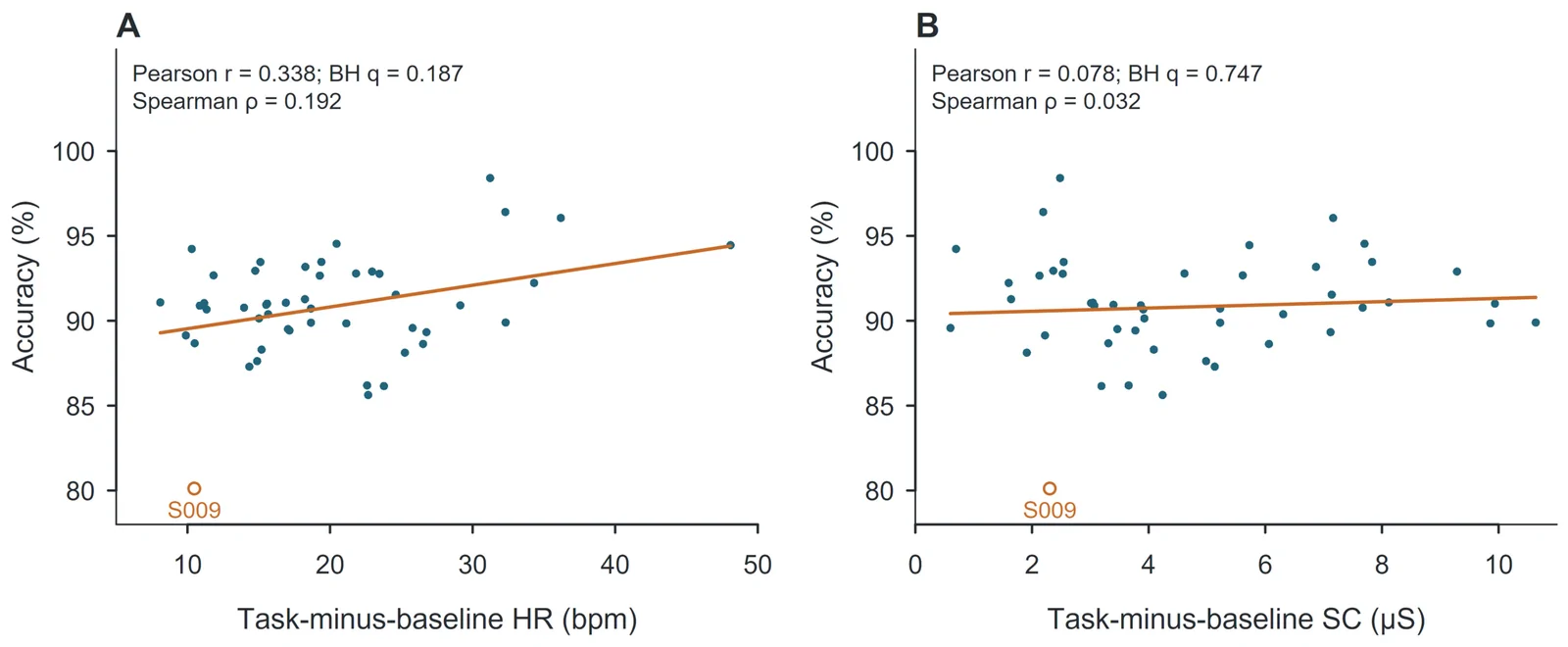
Accuracy in relation to task-minus-baseline HR (**A**) and mean SC (**B**). Blue circles represent participants (*n* = 48); S009, with the lowest accuracy, is an open orange circle. Orange lines show unadjusted OLS fits for display. Annotations give Pearson r, BH q for the ten-test primary family, and Spearman ρ. Accuracy is 100 × correct/(correct + incorrect + omitted responses).

None of the ten primary Pearson correlations met q < 0.05 (Table 4). The largest coefficient was observed for HR change and accuracy: r = 0.338 (95% CI 0.060 to 0.568; *p* = 0.019; q = 0.187). The HR–omission count coefficient was r = −0.291 (95% CI −0.531 to −0.007; *p* = 0.045; q = 0.225). The remaining HR coefficients ranged from −0.209 to 0.181, and mean SC coefficients ranged from −0.092 to 0.078. Figure 2 displays the HR and SC associations with accuracy.

### 4.3. Models Including DT_Stimuli

**Figure 3:**
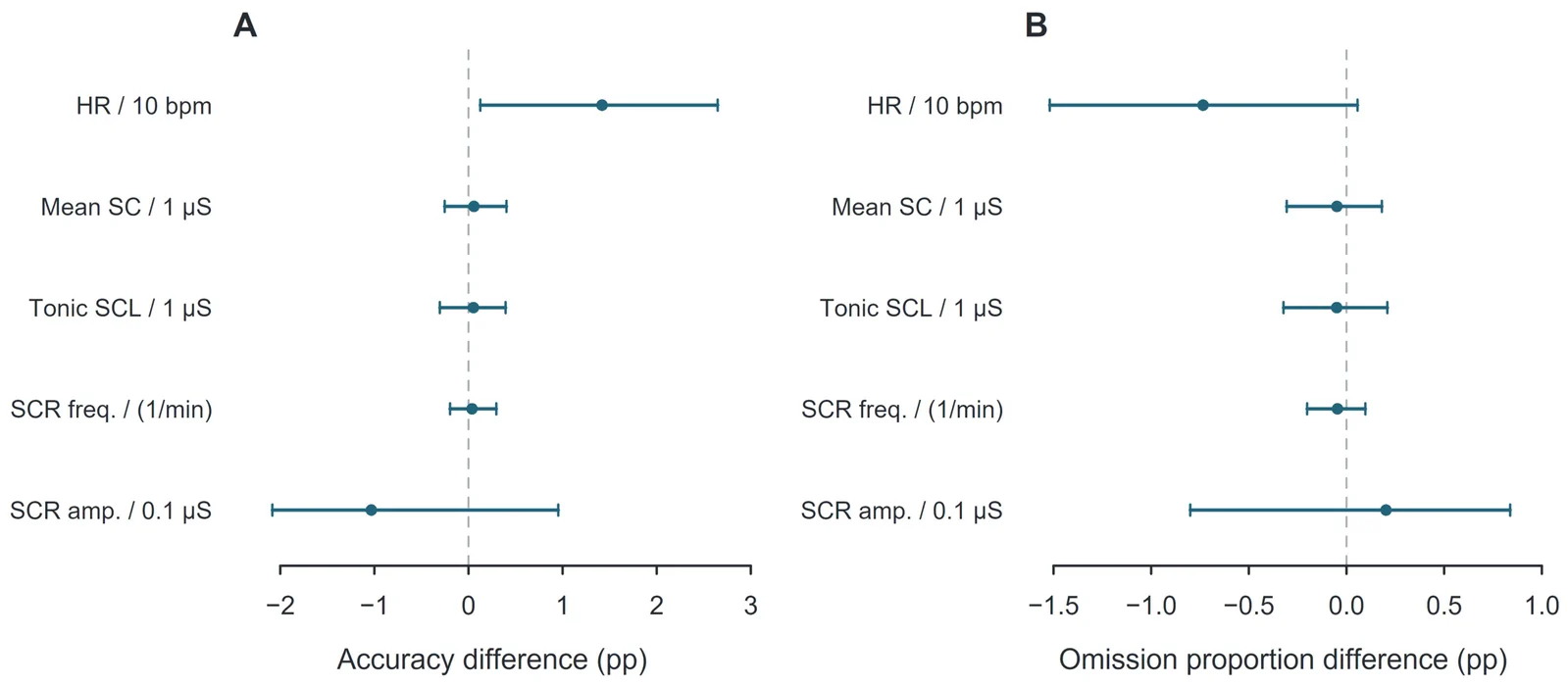
Associations of physiological changes with accuracy (**A**) and omission proportion (**B**) after including corresponding baseline physiology and DT_Stimuli. Points are average marginal derivatives from fractional logit models; lines are individual 95% percentile intervals from 4999 participant bootstrap resamples. Units are stated for each predictor, including 0.1 µS for SCR amplitude. The dashed line marks zero. All displayed effects belong to the 15-test complementary BH family; numerical *p* and q values are in Table 5.

The model including baseline HR and DT_Stimuli estimated an average marginal association with accuracy of 1.42 percentage points per 10 bpm (95% bootstrap CI 0.13 to 2.65; HC3 *p* = 0.033; q = 0.306). The corresponding model without DT_Stimuli estimated 1.37 percentage points. HR estimates were −0.73 percentage points for omission proportion and −0.66 for incorrect response proportion; the median reaction time estimate was 10.55 ms (Table 5). None of the 15 focal model tests met q < 0.05. Estimates for SC, SCL, SCR frequency, and SCR amplitude in relation to accuracy or omissions had bootstrap intervals spanning zero (Figure 3). The HR change-by-baseline interaction coefficient was 0.0009 (95% HC3 CI −0.1662 to 0.1679; q = 0.992).

## 5. Sensitivity Analyses

The HR–accuracy Spearman coefficient was ρ = 0.192 (*p* = 0.190), and the HR–omission coefficient was ρ = −0.215 (*p* = 0.142). Leave-one-out HR–accuracy Pearson coefficients ranged from 0.286 to 0.378. Adjusted fractional logit accuracy effects ranged from 1.05 to 1.60 percentage points per 10 bpm; omitting the participant with the lowest accuracy produced the smallest estimate, 1.05. The unadjusted Theil–Sen accuracy slope was 0.76 percentage points per 10 bpm (95% bootstrap CI −0.50 to 2.20). Effect magnitude therefore varied across analytical specifications and observation spacing. No single deletion reversed the direction of the estimate.

Baseline-adjusted OLS accuracy and omission coefficients both had q = 0.164 after correction across their ten-test family (Table S1). Adding sex and KSS to the fractional-logit HR–accuracy model yielded an effect of 1.40 percentage points per 10 bpm; adding sex and sleep duration yielded 1.37. Replacing DT_Stimuli with DT_Total yielded 1.41. Negative binomial HR incidence rate ratios were 0.852 (95% HC3 CI 0.743 to 0.976) for omissions and 0.841 (0.684 to 1.035) for incorrect responses. These count model estimates were interpreted as sensitivity analyses. Full results are reported in Tables S1–S4.

### Direct Comparison of HR and SC

The HR–SC correlation difference for accuracy was 0.260 (95% BCa CI −0.068 to 0.613; Williams *p* = 0.172; q = 0.599). All five comparison intervals included zero (Table S5). The analyses therefore did not establish stronger HR than SC associations with DT performance.

**Figure 4:**
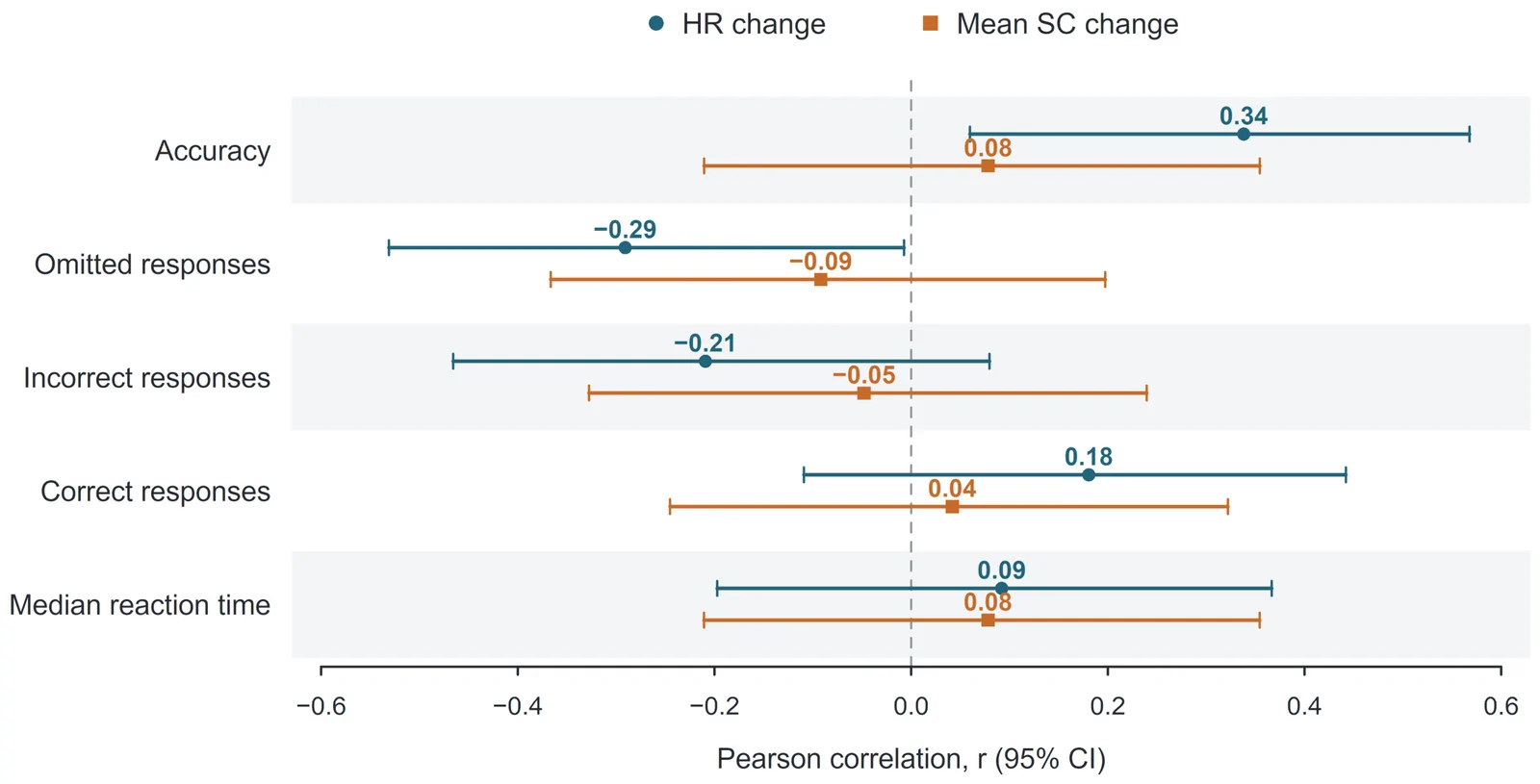
Primary Pearson correlations between task-minus-baseline physiological changes and Determination Test performance. Points show Pearson r and horizontal lines show Fisher-z 95% confidence intervals for *n* = 48. HR (blue circles) and mean SC (orange squares) are shown for accuracy, omitted responses, incorrect responses, correct responses, and median reaction time. The dashed vertical line marks r = 0. Benjamini–Hochberg adjustment was applied across the ten-test primary family; no estimate met q < 0.05. Numerical *p* and q values are reported in Table 4.

Figure 4 summarizes the ten primary Pearson estimates across all five DT outcomes. The two nominal HR intervals that excluded zero correspond to accuracy and omissions; none of the ten tests met the BH-adjusted q < 0.05 threshold.

## 6. Discussion

The transition from seated rest to the adaptive DT was accompanied by increased HR and electrodermal activity, indicating physiological adjustment to the combined cognitive and psychomotor demands of testing. Participants with larger physiological changes did not consistently achieve better behavioral outcomes. The largest primary association was between HR change and accuracy (r = 0.338), but the rank association was smaller (ρ = 0.192), and no primary or complementary model family met the multiplicity-adjusted threshold. The findings therefore distinguish task-related recruitment from reliable differences in performance between individuals.

This distinction helps reconcile the present results with experimental workload research. Mehler et al. found progressive HR and SCL increases as working memory demand increased during driving, even when driving measures did not distinguish successive demand levels [35]. Veltman and Gaillard likewise found that heart period discriminated difficulty levels in a flight task, although several other physiological measures did not [36]. Such findings concern responses to assigned changes in demand. The DT instead continually modifies the demand experienced by each participant. The physiological response can accompany both efforts to maintain performance and the changing demands generated by that performance. The compensatory control framework introduced above helps explain why similar final scores may therefore be reached with different levels of physiological recruitment.

The HR pattern was most apparent for accuracy and omissions. These outcomes are partly linked through the inclusion of omissions in the accuracy denominator. The negative HR–omission coefficient (r = −0.291) was directionally consistent with the positive accuracy estimate, whereas associations with correct response count and median reaction time were weak. This pattern raises a hypothesis about avoiding missed responses rather than a general improvement in speed or response volume. The positive HR–accuracy estimate has a relevant parallel in the work of Magnúsdóttir et al., who reported associations between HR reactivity and performance on digit span and operation span tasks [37]. However, greater cardiac recruitment does not invariably translate into greater success. In repeated video game matches, Behnke et al. found that lower self-reported engagement accompanied blunted HR responses, but neither engagement nor blunted reactivity predicted subsequent performance [38]. Together, these studies suggest that cardiovascular engagement and successful cognition are related but distinct processes. Their expression depends on the cognitive operations required and on whether the outcome captures concurrent performance or performance in a subsequent episode.

Differences between cardiovascular endpoints also matter. Chauntry et al. linked persistence on an unsolvable puzzle primarily to blood pressure reactivity, whereas HR reactivity was associated with persistence during a cold pressor task [39]. Experimental effort research provides a further distinction: Richter et al. observed stronger preejection period and systolic pressure responses as attainable difficulty increased, followed by weaker responses when success became impossible [40]. Monetary incentives also altered preejection period reactivity [41]. These findings support the relationship between motivational investment and cardiac recruitment outlined above, but they do not make mean HR a specific measure of effort or sympathetic drive.

A plausible physiological explanation lies in the multiple routes through which cognitive engagement can change HR. Cardiac acceleration may involve increased sympathetic influence, withdrawal of vagal inhibition, or both [42]. Pharmacological blockade experiments found that psychological stressors produced an overall increase in sympathetic and decrease in parasympathetic cardiac control, yet the contributions of the two branches varied independently across individuals [43]. Similar HR increases can therefore arise from different autonomic combinations, and comparable cognitive demands need not produce identical cardiac responses. Within the compensatory framework introduced above, performance also depends on how effectively the recruited resources are used: additional effort may sustain accuracy as demands rise, while a more efficient strategy may achieve the same outcome with less additional recruitment. These mechanisms offer plausible explanations for an inconsistent association between HR change and performance. The present phase-level measurements do not determine the autonomic contribution or cognitive strategy responsible for an individual’s response.

The DT adds a feedback process to this relationship. Using the adaptive rules described in the Methods, faster correct responses can increase subsequent stimulus presentation, while incorrect and missed reactions slow presentation and reduce later response opportunities. Better performance can therefore generate additional sensory and motor demands within the same test. Including DT_Stimuli changed the HR–accuracy estimate little, but total exposure is itself a result of prior performance. Adjustment for this cumulative count cannot distinguish recruitment that supports responding from recruitment elicited by the ensuing demand. The study-defined accuracy proportion also combines exported response categories rather than mutually exclusive stimulus trials, so its physiological association should be interpreted within that scoring framework.

Baseline cardiac state provides another possible source of heterogeneity. The positive HR estimate remained similar after baseline adjustment, and the interaction analysis provided little evidence of moderation by baseline HR. Nevertheless, baseline HR and vagally mediated variability describe different aspects of cardiac regulation [44,45]. Hansen et al. associated higher resting HRV with better executive task performance [46]; that result cannot be inferred from a low resting HR alone. Accordingly, the present pattern is compatible with several forms of cardiac regulation and does not identify which supported task performance.

Timing offers a further explanation for divergent physiological findings. During prolonged demanding work, Matuz et al. observed decreasing HR and increasing vagally mediated HRV alongside growing fatigue, without evidence of motivational withdrawal from their post-error measures [47]. Hogervorst et al. showed that HR could be sensitive to workload in group analyses yet perform poorly in individual classification when changes over time influenced the signal [48]. Sustained attention itself fluctuates within a task [49]. A phase mean can consequently combine initial recruitment, subsequent adjustment, and local responses to performance events; it need not preserve associations visible at a particular moment or within an individual [50].

The electrodermal findings are consistent with this temporal interpretation. Mean SC and the tonic and phasic summaries increased during DT. Nevertheless, mean SC correlations with the behavioral outcomes were close to zero, and separating tonic SCL, SCR frequency, and SCR amplitude did not yield precise associations with accuracy or omissions. All corresponding adjusted confidence intervals spanned zero. The error-related electrodermal responses and cardiac slowing described in the Introduction provide one possible explanation. Notebaert et al. showed that slowing may also reflect orienting to infrequent events rather than improved control alone [51]. Error-related responses can contribute to electrodermal activity during periods of poorer performance, while sustained engagement may also increase conductance. Combining these contributions over an entire task could weaken the association between phase-level EDA and the final performance score. Neural evidence concerning SCR generation and broader autonomic research also support interpreting response channels in context [52,53]. The direct HR–SC comparisons here did not establish that either signal was more closely associated with performance.

For cognitive assessment, the useful question is therefore whether physiology adds information about the demands under which a behavioral result was achieved. It returns to the clinical motivation outlined in the Introduction. Devos et al., using pupillary responses and P3 potentials in older adults, found that cognitive reserve was associated with aspects of physiological workload [54]. Their findings illustrate why greater activation cannot automatically be equated with better capacity: efficient processing and greater compensatory recruitment may produce different physiological patterns. The present study extends that question to adaptive cognitive and psychomotor testing. Its contribution is to identify the performance–demand relationship that future clinical validation must resolve before physiological summaries can inform individual assessment.

## 7. Future Directions

Future studies should record each stimulus and response alongside synchronized physiological signals, allowing changes in presentation rate to be related to subsequent cardiac and electrodermal responses. A fixed-demand comparison and a yoked condition, in which another participant receives the same stimulus schedule, would help separate individual performance from exposure generated by the adaptive algorithm. Models should distinguish within-person fluctuations from stable differences between participants and evaluate whether physiological changes precede or follow changes in accuracy. ECG-based cardiac measures with recorded respiration could characterize autonomic regulation more precisely, while event-related EDA could distinguish responses to errors from sustained task recruitment. Recording response device activity would improve assessment of motor demands.

Repeated sessions should establish reliability before evaluation in older adults or clinical populations. Such validation should test whether physiology improves prediction beyond behavioral scores and exposure, using prespecified outcomes and independent samples rather than relying on associations within the development dataset.

## 8. Limitations

This exploratory secondary analysis included 48 young adults with self-reported good health. The sample limits the precision of association estimates and their generalizability to clinical populations. The HR–accuracy estimate varied across analytical specifications, and no defined analytical family produced a multiplicity-adjusted finding.

Adaptive exposure developed jointly with performance. Adjustment for baseline physiology and aggregate stimulus count cannot establish whether cardiac changes preceded successful responding or followed the increased sensory and motor demands generated by earlier performance. Correct-plus-incorrect response counts provide only an aggregate proxy for motor activity. Phase summaries also compress temporal variation and cannot identify physiological responses to individual stimuli or errors.

EDA measurements and PPG-based HR tracking are susceptible to motion artifacts [55,56].

The retained preparation records do not document the duration of seated stabilization or meal and hydration requirements.

The available dataset did not include independent measures of cognitive ability, autonomic reactivity to a separate standardized challenge, or enjoyment of cognitively demanding activities. These individual differences may influence the demands participants experience and their willingness to invest effort. Experimental work on cognitive effort discounting has linked the subjective value of demanding task engagement to trait cognitive engagement [57]. Baseline physiological adjustment does not account fully for such differences in capacity, motivation, or autonomic response. Their contribution to the observed physiology–performance associations could not be separated from recruitment during the DT.

## 9. Conclusions

HR and electrodermal measures increased from seated baseline to adaptive DT performance. Mean SC changes were weakly associated with the behavioral outcomes; estimates for the decomposed EDA measures remained uncertain, with confidence intervals spanning zero. A positive HR–accuracy estimate appeared across several specifications, although its magnitude varied, and no defined analytical family produced a multiplicity-adjusted finding. Models incorporating baseline physiology and aggregate stimulus exposure did not distinguish the temporal direction of the performance–demand relationship. Direct comparisons likewise did not establish stronger HR than SC associations. Prospective studies should examine the temporal sequence of stimulus rate, motor responses, performance, and physiology under adaptive and exposure-controlled conditions.

## Figures and Tables

**Table 1:** Participant characteristics.

Characteristic	Value
Women	28 (58.3%)
Men	20 (41.7%)
Age (years)	19.60 ± 1.11
Right-handed	48 (100%)
Height (cm)	174.42 ± 10.64
Body mass (kg)	67.64 ± 14.31
Sleep duration (h)	6.40 ± 1.57
KSS score (1–9)	4.48 ± 1.64

Values are *n* (%) or mean ± SD; *n* = 48. KSS, Karolinska Sleepiness Scale (1–9; higher values indicate greater sleepiness).

**Table 2:** Physiological measurements at baseline and during the DT.

Measure	Baseline Mean ± SD	Task Mean ± SD	Mean Change [95% CI]
HR (bpm)	75.78 ± 10.94	95.77 ± 12.86	19.99 [17.63, 22.35]
Mean SC (µS)	3.99 ± 2.80	8.64 ± 4.18	4.65 [3.92, 5.39]
Tonic SCL (µS)	3.48 ± 2.55	7.85 ± 4.23	4.37 [3.64, 5.11]
SCR frequency (1/min)	5.01 ± 3.20	11.61 ± 4.42	6.60 [5.49, 7.70]
Mean SCR amplitude (µS)	0.180 ± 0.091	0.235 ± 0.088	0.055 [0.026, 0.085]

Baseline and task values are mean ± SD; mean within-participant changes are followed by 95% t-based confidence intervals. HR, heart rate; SC, mean skin conductance; SCL, tonic skin conductance level; SCR, skin conductance response. *n* = 48 throughout.

**Table 3:** Behavioral performance and stimulus exposure during the DT.

DT Outcome	Mean ± SD	Range
Accuracy (%)	90.81 ± 3.08	80.12–98.41
Median RT (ms)	734.62 ± 54.32	641.00–841.00
Correct responses	522.04 ± 45.26	417–596
Incorrect responses	22.69 ± 13.77	3–89
Omitted responses	30.52 ± 11.46	6–54
Response category total	575.25 ± 51.18	483–689
DT_Stimuli	559.08 ± 43.31	474–650

Values are mean ± SD and range; *n* = 48. RT, reaction time. Reaction time summaries describe participant medians. Accuracy is 100 × correct/(correct + incorrect + omitted). DT_Total is the response category total; DT_Stimuli is the number of presented stimuli.

**Table 4:** Primary Pearson correlations with DT outcomes.

Change	Outcome	r [95% CI]	*p*	BH q
ΔHR	Accuracy	0.338 [0.060, 0.568]	0.019	0.187
ΔHR	Omitted count	−0.291 [−0.531, −0.007]	0.045	0.225
ΔHR	Incorrect count	−0.209 [−0.466, 0.080]	0.153	0.512
ΔHR	Correct count	0.181 [−0.109, 0.442]	0.219	0.548
ΔHR	Median RT	0.092 [−0.197, 0.367]	0.534	0.747
ΔSC	Accuracy	0.078 [−0.211, 0.355]	0.597	0.747
ΔSC	Omitted count	−0.092 [−0.367, 0.197]	0.534	0.747
ΔSC	Incorrect count	−0.048 [−0.328, 0.240]	0.747	0.778
ΔSC	Correct count	0.042 [−0.245, 0.322]	0.778	0.778
ΔSC	Median RT	0.078 [−0.211, 0.354]	0.598	0.747

Primary family of ten Pearson correlations, *n* = 48. Δ denotes task minus baseline. Intervals use Fisher-z transformation; *p* values are two-sided and unadjusted, and q values are BH-adjusted across all ten rows. The q < 0.05 threshold was not met by any estimate. Accuracy includes omissions in its denominator.

**Table 5:** Models including baseline physiology and DT_Stimuli.

Change	Outcome	Effect	95% Bootstrap CI	*p*	BH q
ΔHR	Accuracy	1.42	[0.13, 2.65]	0.033	0.306
ΔHR	Omission proportion	−0.73	[−1.52, 0.06]	0.071	0.306
ΔHR	Incorrect proportion	−0.66	[−1.44, 0.09]	0.082	0.306
ΔHR	Median RT	10.55	[−0.004, 21.126]	0.048	0.306
ΔSC	Accuracy	0.06	[−0.25, 0.40]	0.724	0.891
ΔSC	Omission proportion	−0.05	[−0.31, 0.18]	0.694	0.891
ΔSC	Incorrect proportion	−0.01	[−0.20, 0.16]	0.949	0.992
ΔSC	Median RT	2.01	[−2.716, 7.017]	0.492	0.891
ΔSCL	Accuracy	0.05	[−0.30, 0.39]	0.763	0.891
ΔSCL	Omission proportion	−0.05	[−0.32, 0.21]	0.725	0.891
ΔSCR frequency	Accuracy	0.04	[−0.20, 0.30]	0.772	0.891
ΔSCR frequency	Omission proportion	−0.05	[−0.20, 0.10]	0.567	0.891
ΔSCR amplitude	Accuracy	−1.03	[−2.08, 0.95]	0.368	0.891
ΔSCR amplitude	Omission proportion	0.20	[−0.80, 0.84]	0.662	0.891

*n* = 48. Each additive model included the physiological change shown, its corresponding baseline value, and DT_Stimuli/100. Proportion outcomes were analyzed with fractional logit and equal participant weights; RT was analyzed with OLS. Effects are average marginal derivatives in percentage points (pp), except for RT slopes in milliseconds. Intervals are 95% participant bootstrap percentile intervals based on 4999 resamples. The p values use HC3 t-based inference for the focal coefficient. BH q values cover the 14 displayed effects and the HR change-by-baseline interaction reported in the text. Predictor units are 10 bpm for HR and 1 µS for SC or SCL. Units are 1/min for SCR frequency and 0.1 µS for SCR amplitude. Confidence intervals are individual rather than simultaneous.

## Data Availability

The statistical analysis results are provided in Supplementary File S2. Additional statistical results are presented in Supplementary File S3 (Tables S1–S6 and Figures S1 and S2).

## Supplementary Materials

The following supporting information can be downloaded at: https://www.mdpi.com/article/10.3390/jcm15208068/s1. Supplementary File S1 contains the ethics approval (no. 46/2025, signed 28 January 2026); Supplementary File S2: Reproducible statistical analyses. The statistical analysis results, including models with sex, KSS, and sleep duration, are provided in Supplementary File S2; Supplementary File S3: Supplementary Table S1: Baseline adjusted OLS sensitivity analyses; Table S2 Rank robust slope and leave one out analyses; Table S3 Sensitivity of the HR; Table S4 Negative binomial models with DT_Stimuli offset; Table S5 Direct comparison of HR and SC correlations; Table S6 HR associations with aggregate exposure summaries; Supplementary Figures S1 and S2 present influence and residual diagnostics. These tables and figures are in Supplementary File S3. Figure S1. Pearson HR–accuracy leave-one-out estimates (left) and Cook’s distance for the accuracy fractional-logit model with baseline HR and DT_Stimuli (right). The horizontal Cook’s-distance line is 4/n, used as a screening reference rather than an exclusion rule; Figure S2. Residual displays for the accuracy fractional-logit model (left) and median-RT OLS model (right), each containing HR change, baseline HR, and DT_Stimuli. The smooth curves show descriptive residual patterns.

## References

[1] Hockey G.R.J. (1997). Compensatory control in the regulation of human performance under stress and high workload: A cognitive-energetical framework. Biol. Psychol..

[2] Forstmann B.U., Dutilh G., Brown S., Neumann J., von Cramon D.Y., Ridderinkhof K.R., Wagenmakers E.J. (2008). Striatum and pre-SMA facilitate decision-making under time pressure. Proc. Natl. Acad. Sci. USA.

[3] Ratcliff R., McKoon G. (2008). The Diffusion Decision Model: Theory and Data for Two-Choice Decision Tasks. Neural Comput..

[4] Beissner F., Meissner K., Bär K.J., Napadow V. (2013). The Autonomic Brain: An Activation Likelihood Estimation Meta-Analysis for Central Processing of Autonomic Function. J. Neurosci..

[5] Critchley H.D., Mathias C.J., Josephs O., O’Doherty J., Zanini S., Dewar B.K., Cipolotti L., Shallice T., Dolan R.J. (2003). Human cingulate cortex and autonomic control: Converging neuroimaging and clinical evidence. Brain.

[6] Charles R.L., Nixon J. (2019). Measuring mental workload using physiological measures: A systematic review. Appl. Ergon..

[7] Alsanousi M.M., Prabhu V.V. (2026). Non-Invasive Physiological Metrics for Cognitive Load Assessment in Training and Operational Contexts: Signal Processing, Evidence, and Feasibility. Sensors.

[8] Hughes A.M., Hancock G.M., Marlow S.L., Stowers K., Salas E. (2019). Cardiac Measures of Cognitive Workload: A Meta-Analysis. Hum. Factors.

[9] Gendolla G.H.E. (2025). The intensity of mental effort: “the heart does not lie”. Synthese.

[10] Berntson G.G., Cacioppo J.T., Quigley K.S. (1991). Autonomic determinism: The modes of autonomic control, the doctrine of autonomic space, and the laws of autonomic constraint. Psychol. Rev..

[11] Boucsein W., Fowles D.C., Grimnes S., Ben-Shakhar G., Roth W.T., Dawson M.E., Filion D.L. (2012). Publication recommendations for electrodermal measurements. Psychophysiology.

[12] Macefield V.G., Wallin B.G. (1996). The discharge behaviour of single sympathetic neurones supplying human sweat glands. J. Auton. Nerv. Syst..

[13] Benedek M., Kaernbach C. (2010). A continuous measure of phasic electrodermal activity. J. Neurosci. Methods.

[14] Conklin S., Kargosha G., Tu J., Bansal K., Dang Q., Brooks J., Kucukosmanoglu M. (2026). Cognitive biotypes identified through ECG-derived workload and behavioral accuracy. Sci. Rep..

[15] Hajcak G., McDonald N., Simons R.F. (2003). To err is autonomic: Error-related brain potentials, ANS activity, and post-error compensatory behavior. Psychophysiology.

[16] SCHUHFRIED DT Determination Test. https://marketplace.schuhfried.com/en/DT.

[17] Tao D., Tan H., Wang H., Zhang X., Qu X., Zhang T. (2019). A Systematic Review of Physiological Measures of Mental Workload. Int. J. Environ. Res. Public Health.

[18] Fisher A.J., Medaglia J.D., Jeronimus B.F. (2018). Lack of group-to-individual generalizability is a threat to human subjects research. Proc. Natl. Acad. Sci. USA.

[19] Hedge C., Powell G., Sumner P. (2018). The reliability paradox: Why robust cognitive tasks do not produce reliable individual differences. Behav. Res. Methods.

[20] Ranchet M., Morgan J.C., Akinwuntan A.E., Devos H. (2017). Cognitive workload across the spectrum of cognitive impairments: A systematic review of physiological measures. Neurosci. Biobehav. Rev..

[21] von Elm E., Altman D.G., Egger M., Pocock S.J., Gøtzsche P.C., Vandenbroucke J.P. (2007). The Strengthening the Reporting of Observational Studies in Epidemiology (STROBE) Statement: Guidelines for Reporting Observational Studies. PLoS Med..

[22] Allen J. (2007). Photoplethysmography and its application in clinical physiological measurement. Physiol. Meas..

[23] Greco A., Valenza G., Lanata A., Scilingo E.P., Citi L. (2016). cvxEDA: A Convex Optimization Approach to Electrodermal Activity Processing. IEEE Trans. Biomed. Eng..

[24] Kleckner I.R., Jones R.M., Wilder-Smith O., Wormwood J.B., Akcakaya M., Quigley K.S., Lord C., Goodwin M.S. (2018). Simple, Transparent, and Flexible Automated Quality Assessment Procedures for Ambulatory Electrodermal Activity Data. IEEE Trans. Biomed. Eng..

[25] SCHUHFRIED (2023). Determination Test (DT): Manual, Version 51, Revision 4.

[26] de Winter J.C.F., Gosling S.D., Potter J. (2016). Comparing the Pearson and Spearman correlation coefficients across distributions and sample sizes: A tutorial using simulations and empirical data. Psychol. Methods.

[27] Benjamini Y., Hochberg Y. (1995). Controlling the False Discovery Rate: A Practical and Powerful Approach to Multiple Testing. J. R. Stat. Soc. Ser. B.

[28] Wasserstein R.L., Lazar N.A. (2016). The ASA Statement on p-Values: Context, Process, and Purpose. Am. Stat..

[29] MacKinnon J.G., White H. (1985). Some heteroskedasticity-consistent covariance matrix estimators with improved finite sample properties. J. Econom..

[30] Papke L.E., Wooldridge J.M. (1996). Econometric methods for fractional response variables with an application to 401(k) plan participation rates. J. Appl. Econom..

[31] Sen P.K. (1968). Estimates of the Regression Coefficient Based on Kendall’s Tau. J. Am. Stat. Assoc..

[32] Cook R.D. (1977). Detection of Influential Observation in Linear Regression. Technometrics.

[33] Diedenhofen B., Musch J. (2015). cocor: A Comprehensive Solution for the Statistical Comparison of Correlations. PLoS ONE.

[34] Efron B. (1987). Better Bootstrap Confidence Intervals. J. Am. Stat. Assoc..

[35] Mehler B., Reimer B., Coughlin J.F. (2012). Sensitivity of Physiological Measures for Detecting Systematic Variations in Cognitive Demand From a Working Memory Task: An On-Road Study Across Three Age Groups. Hum. Factors.

[36] Veltman J.A., Gaillard A.W.K. (1998). Physiological workload reactions to increasing levels of task difficulty. Ergonomics.

[37] Magnúsdóttir B.B., Gylfason H.F., Jóhannsdóttir K.R. (2023). Blunted Cardiovascular Reactivity Predicts Worse Performance in Working Memory Tasks. Brain Sci..

[38] Behnke M., Hase A., Kaczmarek L.D., Freeman P. (2021). Blunted cardiovascular reactivity may serve as an index of psychological task disengagement in the motivated performance situations. Sci. Rep..

[39] Chauntry A.J., Williams S.E., Whittaker A.C. (2019). Blunted cardiovascular responses to acute psychological stress predict low behavioral but not self-reported perseverance. Psychophysiology.

[40] Richter M., Friedrich A., Gendolla G.H.E. (2008). Task difficulty effects on cardiac activity. Psychophysiology.

[41] Richter M., Gendolla G.H.E. (2009). The heart contracts to reward: Monetary incentives and preejection period. Psychophysiology.

[42] Quigley K.S., Gianaros P.J., Norman G.J., Jennings J.R., Berntson G.G., de Geus E.J.C. (2024). Publication guidelines for human heart rate and heart rate variability studies in psychophysiology—Part 1: Physiological underpinnings and foundations of measurement. Psychophysiology.

[43] Berntson G.G., Cacioppo J.T., Binkley P.F., Uchino B.N., Quigley K.S., Fieldstone A. (1994). Autonomic cardiac control. III. Psychological stress and cardiac response in autonomic space as revealed by pharmacological blockades. Psychophysiology.

[44] Laborde S., Mosley E., Thayer J.F. (2017). Heart Rate Variability and Cardiac Vagal Tone in Psychophysiological Research–Recommendations for Experiment Planning, Data Analysis, and Data Reporting. Front. Psychol..

[45] Berntson G.G., Uchino B.N., Cacioppo J.T. (1994). Origins of baseline variance and the Law of Initial Values. Psychophysiology.

[46] Hansen A.L., Johnsen B.H., Thayer J.F. (2003). Vagal influence on working memory and attention. Int. J. Psychophysiol..

[47] Matuz A., van der Linden D., Kisander Z., Hernádi I., Karádi K., Csathó Á. (2021). Enhanced cardiac vagal tone in mental fatigue: Analysis of heart rate variability in Time-on-Task, recovery, and reactivity. PLoS ONE.

[48] Hogervorst M.A., Brouwer A.M., van Erp J.B.F. (2014). Combining and comparing EEG, peripheral physiology and eye-related measures for the assessment of mental workload. Front. Neurosci..

[49] Esterman M., Noonan S.K., Rosenberg M., DeGutis J. (2013). In the Zone or Zoning Out? Tracking Behavioral and Neural Fluctuations During Sustained Attention. Cereb. Cortex.

[50] Curran P.J., Bauer D.J. (2011). The Disaggregation of Within-Person and Between-Person Effects in Longitudinal Models of Change. Annu. Rev. Psychol..

[51] Notebaert W., Houtman F., Van Opstal F., Gevers W., Fias W., Verguts T. (2009). Post-error slowing: An orienting account. Cognition.

[52] Critchley H.D., Elliott R., Mathias C.J., Dolan R.J. (2000). Neural Activity Relating to Generation and Representation of Galvanic Skin Conductance Responses: A Functional Magnetic Resonance Imaging Study. J. Neurosci..

[53] Kreibig S.D. (2010). Autonomic nervous system activity in emotion: A review. Biol. Psychol..

[54] Devos H., Gustafson K.M., Liao K., Ahmadnezhad P., Kuhlmann E., Estes B.J., Martin L.E., Mahnken J.D., Brooks W.M., Burns J.M. (2023). Effect of Cognitive Reserve on Physiological Measures of Cognitive Workload in Older Adults with Cognitive Impairments. J. Alzheimer’s Dis..

[55] Taylor S., Jaques N., Chen W., Fedor S., Sano A., Picard R. Automatic identification of artifacts in electrodermal activity data. Proceedings of the 2015 37th Annual International Conference of the IEEE Engineering in Medicine and Biology Society (EMBC).

[56] Ismail S., Akram U., Siddiqi I. (2021). Heart rate tracking in photoplethysmography signals affected by motion artifacts: A review. EURASIP J. Adv. Signal Process..

[57] Westbrook A., Kester D., Braver T.S. (2013). What Is the Subjective Cost of Cognitive Effort? Load, Trait, and Aging Effects Revealed by Economic Preference. PLoS ONE.

