# Supplementary material for: Autonomic Changes During an Adaptive Cognitive Task
Source: J Clin Med. 2026 Oct 17;15(20):8068. doi: 10.3390/jcm15208068 (PMC13705764; doi:10.3390/jcm15208068)
Supplement: Supplementary file 1 [file jcm-15-08068-s001.zip › Supplementary_File_S2_Reanalysis.pdf]

JCM 4596147 | Reproducible statistical analyses

Reproducible analyses from Wyniki_Nowe.xlsx (48 participants).
Sections 01-09 contain the completed statistical analysis outputs.
Analysis results are provided in Supplementary File S2.
Bootstrap seed 4596147 is reset per model. No raw-signal EDA processing.
The original native analyses follow; conventional OLS SE are reference results only.
Sex, KSS and sleep duration come from the original OMV, joined by Subject_ID.
The accompanying updated supplement uses these reproducible S2/S5 bootstrap intervals.

01 | Excel source, descriptives and EDA phase checks

Source: Wyniki_Nowe.xlsx / Participant_results
n = 48 ; complete required analysis variables. No participant exclusions.

Descriptive statistics (source units)

variable	n	mean	SD	median	min	max
DT_Correct	48	522.04166667	45.26374639	523.00000000	417.00000000	596.0000000
DT_Incorrect	48	22.68750000	13.77348092	21.00000000	3.00000000	89.0000000
DT_Omitted	48	30.52083333	11.46129394	31.50000000	6.00000000	54.0000000
DT_Total	48	575.25000000	51.17782931	567.50000000	483.00000000	689.0000000
DT_Stimuli	48	559.08333333	43.30634730	554.00000000	474.00000000	650.0000000
DT_Accuracy	48	0.90810624	0.03076013	0.90899680	0.80116110	0.9840708
DT_Omission_proportion	48	0.05344791	0.02064517	0.05415741	0.01061947	0.1006160
DT_RT_median_s	48	0.73462500	0.05431924	0.73550000	0.64100000	0.8410000
HR_Baseline_bpm	48	75.77746287	10.93791602	76.64650000	48.98700000	100.0920000
HR_Task_bpm	48	95.76658146	12.86322847	94.82877750	74.25900000	121.9233410
HR_Change_bpm	48	19.98911858	8.12672227	18.46185800	8.09400000	48.0933410
SC_Baseline_uS	48	3.98809485	2.79783187	3.52100000	0.62045900	12.3220000
SC_Task_uS	48	8.64206392	4.18352606	7.40600000	2.51090100	17.3263800
SC_Change_uS	48	4.65396906	2.54114713	3.91709050	0.60200000	10.6370000
Baseline_SCL_uS	48	3.48082136	2.55133100	3.09856114	0.58975516	11.3549641
Task_SCL_uS	48	7.85459607	4.23261344	6.81526447	1.27724603	16.9753046
Delta_SCL_uS	48	4.37377470	2.52070436	3.67299889	0.59699332	10.1386638
Baseline_SCR_count_n	48	24.68750000	15.78196688	22.50000000	1.00000000	67.0000000
Task_SCR_count_n	48	94.20833333	35.83885822	97.00000000	9.00000000	180.0000000
Baseline_SCR_model_per_min	48	5.01474564	3.20032219	4.61840731	0.20516636	13.6051414
Task_SCR_model_per_min	48	11.60982512	4.42424066	12.05649884	1.10514198	22.7368421
Delta_SCR_model_per_min	48	6.59507948	3.81960282	6.41501971	0.08363852	14.8197697
Baseline_SCR_amplitude_zero_coded_uS	48	0.17954049	0.09058020	0.17216631	0.05593884	0.4699255
Task_SCR_amplitude_zero_coded_uS	48	0.23503806	0.08797837	0.22548713	0.06221050	0.5000236
Delta_SCR_amplitude_zero_coded_uS	48	0.05549758	0.10007377	0.05281245	-0.21616237	0.4150962
Baseline_EDA_model_exposure_s	48	295.44114925	5.67351922	293.33533997	286.25000000	315.6250000
Baseline_phase_duration_s	48	304.10173055	5.88610133	301.57000317	296.16944458	325.5230320
Task_EDA_model_exposure_s	48	487.23778197	15.00510924	489.18750000	392.75000000	506.7500000
Task_phase_duration_s	48	497.94417689	14.41913470	499.25256450	402.76542383	516.7520710

Mean changes: participant t intervals (HR in bpm)

component	mean_change	SD	CI_low	CI_high
HR	19.98911858	8.1267223	17.62936442	22.34887275
SC	4.65396906	2.5411471	3.91609685	5.39184127
SCL	4.37377470	2.5207044	3.64183845	5.10571095
SCRfreq	6.59507948	3.8196028	5.48598243	7.70417652
SCRamp	0.05549758	0.1000738	0.02643918	0.08455597

Exposure and count checks; flags do not remove records

Subject_ID	total_minus_stimuli	correct_exceeds_stimuli	baseline_exposure_s	baseline_duration_s	baseline_coverage_pct	task_exposure_s
S001	17	FALSE	291.4407	300.6907	96.92375	486.5000
S002	5	FALSE	303.3750	313.3739	96.80928	491.5000
S003	21	FALSE	295.4765	302.7265	97.60510	486.8750
S004	55	TRUE	300.2500	310.1540	96.80675	492.5000
S005	10	FALSE	315.6250	325.5230	96.95935	489.0000
S006	6	FALSE	293.1435	301.5185	97.22239	487.5000
S007	10	FALSE	294.4824	302.2324	97.43575	490.2500
S008	10	FALSE	293.2266	301.6016	97.22316	490.0000
S009	76	FALSE	291.9021	300.9021	97.00899	487.8750
S010	9	FALSE	292.1826	301.0576	97.05206	493.2500
S011	41	FALSE	301.4166	301.4166	100.00000	500.3843
S012	41	FALSE	292.1491	301.0241	97.05173	491.1250
S013	11	FALSE	301.7685	301.7685	100.00000	499.2792
S014	6	FALSE	291.3782	300.6282	96.92311	475.0000
S015	21	FALSE	291.3786	300.6286	96.92311	489.0000
S016	5	FALSE	304.8750	314.8372	96.83577	487.6250
S017	24	FALSE	293.4441	301.6941	97.26544	477.7500
S018	8	FALSE	291.9207	300.9207	97.00918	491.1250
S019	7	FALSE	293.7474	301.8724	97.30847	490.5000
S020	38	FALSE	291.1600	300.5350	96.88056	477.7500
S021	10	FALSE	290.4563	300.2063	96.75223	494.1250
S022	9	FALSE	295.6250	305.5411	96.75457	490.7500
S023	12	FALSE	286.2500	296.1694	96.65075	490.5000
S024	12	FALSE	292.2466	301.1216	97.05269	506.7500
S025	33	FALSE	292.1721	301.0471	97.05196	477.1250
S026	7	FALSE	291.7214	300.8464	96.96689	489.1250
S027	14	FALSE	299.6250	309.5340	96.79873	489.8750
S028	6	FALSE	293.1634	301.5384	97.22258	493.1250
S029	30	FALSE	295.6413	302.7663	97.64670	490.6250
S030	13	FALSE	293.6547	301.7797	97.30764	487.7500
S031	3	FALSE	291.9580	300.9580	97.00955	490.3750
S032	8	FALSE	296.0000	305.8991	96.76395	489.3750
S033	4	FALSE	291.2386	300.6136	96.88138	487.2500
S034	21	FALSE	290.4928	300.2428	96.75263	485.5000
S035	11	FALSE	313.2500	323.2218	96.91487	489.0000
S036	9	FALSE	298.3750	308.3453	96.76653	491.3750
S037	10	FALSE	292.6824	301.3074	97.13748	488.2500
S038	13	FALSE	292.7240	301.3490	97.13787	489.2500
S039	4	FALSE	293.6453	301.7703	97.30755	479.7500
S040	24	FALSE	299.8750	309.7925	96.79868	498.6250
S041	6	FALSE	291.4919	300.7419	96.92427	488.8750
S042	15	FALSE	303.6250	313.6205	96.81286	392.7500
S043	19	FALSE	292.4502	301.2002	97.09496	489.3750
S044	16	FALSE	295.9632	302.9632	97.68949	488.7500

```
       S045                15                FALSE           295.3750           305.2793             96.75566        489.6250
       S046                 2                FALSE           292.4456           301.1956             97.09491        489.1250
       S047                12                FALSE           293.6848           301.8098             97.30791        488.6250
       S048                17                FALSE           301.0000           310.9168             96.81047        487.0000
 task_duration_s task_coverage_pct
        496.5008          97.98575
        501.5767          97.99100
        496.9020          97.98209
        502.5295          98.00420
        499.0612          97.98397
        497.4837          97.99316
        500.2620          97.99864
        499.9903          98.00191
        497.9436          97.97797
        503.2924          98.00466
        500.3843         100.00000
        501.1144          98.00656
        499.2792         100.00000
        497.5178          95.47397
        498.9917          97.99761
        497.6830          97.97904
        497.8579          95.96112
        501.1351          98.00252
        500.5675          97.98879
        498.3096          95.87413
        504.1393          98.01359
        500.6682          98.01901
        500.5301          97.99610
        516.7521          98.06444
        497.1296          95.97598
        499.1557          97.99046
        499.8846          97.99762
        503.1411          98.00930
        500.6297          98.00157
        497.7503          97.99091
        500.3100          98.01423
        499.4454          97.98368
        497.2557          97.98781
        495.5906          97.96392
        499.0641          97.98340
        501.4595          97.98897
        498.3559          97.97216
        499.1972          98.00736
        499.7312          96.00161
        508.5125          98.05561
        498.9342          97.98387
        402.7654          97.51334
        499.3177          98.00873
        498.7310          97.99872
        499.6105          98.00134
        499.2259          97.97668
        498.5608          98.00709
        497.0894          97.97030
```

```
Phase coverage is exposure/duration from the workbook; it is not independent proof of raw-signal cleaning.
```

```
Task-minus-baseline reconciliation
 component max_change_discrepancy
        HR           1.421085e-14
        SC           1.776357e-15
       SCL           0.000000e+00
   SCRfreq           0.000000e+00
    SCRamp           0.000000e+00
```

02 | Table 4 Pearson and Table S1 OLS-HC3

```
Table 4: Pearson correlations; Fisher-z CI; BH family of 10
 change      outcome  n           r      CI_low      CI_high          p   q_BH_10
     HR Accuracy_pct 48  0.33817651  0.05978656  0.567756773 0.01872674 0.1872674
     HR   DT_Omitted 48 -0.29079273 -0.53104981 -0.007257696 0.04494948 0.2247474
     HR DT_Incorrect 48 -0.20924178 -0.46568989  0.079626963 0.15349012 0.5116337
     HR   DT_Correct 48  0.18064218 -0.10909185  0.442086052 0.21919411 0.5479853
     HR     DT_RT_ms 48  0.09206541 -0.19722859  0.366609967 0.53370625 0.7470389
     SC Accuracy_pct 48  0.07825163 -0.21056477  0.354504277 0.59703838 0.7470389
     SC   DT_Omitted 48 -0.09195306 -0.36651188  0.197337483 0.53420777 0.7470389
     SC DT_Incorrect 48 -0.04788352 -0.32756162  0.239509801 0.74655329 0.7777595
     SC   DT_Correct 48  0.04182244 -0.24522634  0.322129208 0.77775950 0.7777595
     SC     DT_RT_ms 48  0.07812559 -0.21068596  0.354393392 0.59763115 0.7470389
```

```
Table S1: baseline-adjusted OLS; HC3 t inference; BH family of 10
 change      outcome  n    estimate    SE_HC3 df      CI_low    CI_high          p   q_BH_10
     HR Accuracy_pct 48  1.29753040 0.5634133 45   0.1627578  2.4323030 0.02595717 0.1641733
     HR   DT_Omitted 48 -4.12663558 1.8740654 45  -7.9011970 -0.3520742 0.03283465 0.1641733
     HR DT_Incorrect 48 -3.47325381 2.6679919 45  -8.8468653  1.9003577 0.19960170 0.4990043
     HR   DT_Correct 48 11.33229159 6.7524351 45  -2.2678108 24.9323940 0.10023050 0.3341017
     HR     DT_RT_ms 48  4.42652489 9.7681692 45 -15.2475778 24.1006276 0.65261250 0.8077584
     SC Accuracy_pct 48  0.05624502 0.1600930 45  -0.2661988  0.3786888 0.72698255 0.8077584
     SC   DT_Omitted 48 -0.32226766 0.6629267 45  -1.6574706  1.0129352 0.62923495 0.8077584
     SC DT_Incorrect 48 -0.08465102 0.7335258 45  -1.5620478  1.3927458 0.90863920 0.9086392
     SC   DT_Correct 48  1.11284690 2.4687813 45  -3.8595339  6.0852277 0.65432155 0.8077584
     SC     DT_RT_ms 48  1.51926976 3.7427480 45  -6.0190116  9.0575512 0.68672254 0.8077584
```

```
Slope units: HR per 10 bpm; SC per 1 uS. Accuracy in percentage points; RT in ms.
```

```
All OLS coefficients, including intercept and baseline; p values unadjusted
           model            term     estimate     SE_HC3 df       CI_low      CI_high            p
 HR Accuracy_pct     (Intercept)  87.35005970  3.2491940 45  80.80584706  93.89427234 2.320461e-29
 HR Accuracy_pct      Delta_HR_10   1.29753040  0.5634133 45   0.16275783   2.43230297 2.595717e-02
 HR Accuracy_pct HR_Baseline_bpm   0.01144029  0.0358172 45  -0.06069926   0.08357983 7.508956e-01
   HR DT_Omitted     (Intercept)  40.03306712 12.0911841 45  15.68017217  64.38596206 1.838414e-03
   HR DT_Omitted      Delta_HR_10  -4.12663558  1.8740654 45  -7.90119697  -0.35207419 3.283465e-02
   HR DT_Omitted HR_Baseline_bpm  -0.01667320  0.1384882 45  -0.29560268   0.26225628 9.047067e-01
 HR DT_Incorrect     (Intercept)  26.01363693 13.9692276 45  -2.12183175  54.14910560 6.911071e-02
 HR DT_Incorrect      Delta_HR_10  -3.47325381  2.6679919 45  -8.84686529   1.90035767 1.996017e-01
```

```
 HR DT_Incorrect HR_Baseline_bpm   0.04772648  0.1509807 45  -0.25636430   0.35181725 7.533808e-01
   HR DT_Correct     (Intercept) 436.47019193 50.2249365 45 335.31197718 537.62840668 3.486601e-11
   HR DT_Correct     Delta_HR_10  11.33229159  6.7524351 45  -2.26781084  24.93239401 1.002305e-01
   HR DT_Correct HR_Baseline_bpm   0.83031577  0.6059123 45  -0.39005428   2.05068583 1.773735e-01
     HR DT_RT_ms     (Intercept) 811.27803754 59.7842071 45 690.86646339 931.68961169 1.632467e-17
     HR DT_RT_ms     Delta_HR_10   4.42652489  9.7681692 45 -15.24757784  24.10062762 6.526125e-01
     HR DT_RT_ms HR_Baseline_bpm  -1.12832058  0.7444798 45  -2.62777990   0.37113874 1.366185e-01
 SC Accuracy_pct     (Intercept)  89.93274726  1.2280421 45  87.45934349  92.40615104 1.899254e-48
 SC Accuracy_pct     SC_Change_uS   0.05624502  0.1600930 45  -0.26619878   0.37868882 7.269826e-01
 SC Accuracy_pct  SC_Baseline_uS   0.15448845  0.1564442 45  -0.16060636   0.46958325 3.286784e-01
   SC DT_Omitted     (Intercept)  33.50127737  3.9139411 45  25.61819537  41.38435936 5.363426e-11
   SC DT_Omitted     SC_Change_uS  -0.32226766  0.6629267 45  -1.65747055   1.01293522 6.292350e-01
   SC DT_Omitted  SC_Baseline_uS  -0.37126005  0.7045946 45  -1.79038636   1.04786627 6.008420e-01
 SC DT_Incorrect     (Intercept)  25.88182553  6.3645226 45  13.06301904  38.70063202 1.895005e-04
 SC DT_Incorrect     SC_Change_uS  -0.08465102  0.7335258 45  -1.56204784   1.39274580 9.086392e-01
 SC DT_Incorrect  SC_Baseline_uS  -0.70218046  0.8453441 45  -2.40479085   1.00042993 4.105600e-01
   SC DT_Correct     (Intercept) 522.75336916 15.3279724 45 491.88124794 553.62549038 8.523498e-34
   SC DT_Correct     SC_Change_uS   1.11284690  2.4687813 45  -3.85953391   6.08522771 6.543215e-01
   SC DT_Correct  SC_Baseline_uS  -1.47711069  3.1588770 45  -7.83941561   4.88519423 6.423229e-01
     SC DT_RT_ms     (Intercept) 725.14076020 19.4210555 45 686.02474650 764.25677391 1.658237e-35
     SC DT_RT_ms     SC_Change_uS   1.51926976  3.7427480 45  -6.01901165   9.05755118 6.867225e-01
     SC DT_RT_ms  SC_Baseline_uS   0.60520258  4.2380907 45  -7.93075017   9.14115534 8.870850e-01
```

03 | Table 5 fractional logit, OLS, interaction and bootstrap

```
Table 5: additive models, HC3 p and 4999 paired-participant percentile intervals
  change                outcome  n       effect bootstrap_low bootstrap_high focal_coefficient     SE_HC3 df          p converged    B    q_BH_15
      HR            DT_Accuracy 48  1.419034445   0.125351840     2.64774030       0.170304071 0.07757221 44 0.03345192      4999 4999 0.3061551
      HR DT_Omission_proportion 48 -0.734295352  -1.519385797     0.05648643      -0.145307228 0.07849958 44 0.07088095      4999 4999 0.3061551
      HR   Incorrect_proportion 48 -0.660565940  -1.435326729     0.09011639      -0.179156480 0.10053339 44 0.08164135      4999 4999 0.3061551
      HR               DT_RT_ms 48 10.550067359  -0.004000611    21.12585598      10.550067359 5.17465491 44 0.04750638      4999 4999 0.3061551
      SC            DT_Accuracy 48  0.057529848  -0.253045317     0.40337977       0.006896408 0.01937929 44 0.72364236      4999 4999 0.8911091
      SC DT_Omission_proportion 48 -0.049087115  -0.306144193     0.18092067      -0.009709125 0.02452440 44 0.69409459      4999 4999 0.8911091
      SC   Incorrect_proportion 48 -0.006159353  -0.203997845     0.16388429      -0.001669307 0.02578650 44 0.94867768      4999 4999 0.9917336
      SC               DT_RT_ms 48  2.008993314  -2.715949419     7.01684149       2.008993314 2.89765258 44 0.49175415      4999 4999 0.8911091
     SCL            DT_Accuracy 48  0.052906370  -0.304466776     0.39400990       0.006342254 0.02093466 44 0.76335215      4999 4999 0.8911091
     SCL DT_Omission_proportion 48 -0.050065186  -0.322586918     0.20913625      -0.009904654 0.02797478 44 0.72498759      4999 4999 0.8911091
 SCRfreq            DT_Accuracy 48  0.037251233  -0.195914343     0.29565772       0.004465339 0.01533620 44 0.77229457      4999 4999 0.8911091
 SCRfreq DT_Omission_proportion 48 -0.046481964  -0.201532202     0.09647739      -0.009193664 0.01593077 44 0.56681243      4999 4999 0.8911091
  SCRamp            DT_Accuracy 48 -1.032602384  -2.084370446     0.95353171      -1.238684703 1.36121621 44 0.36778919      4999 4999 0.8911091
  SCRamp DT_Omission_proportion 48  0.202166244  -0.799832008     0.83801496       0.399843437 0.90840494 44 0.66197603      4999 4999 0.8911091

Equal participant weights. Proportion effects are average marginal derivatives in percentage points.
HR unit: 10 bpm; SC/SCL: 1 uS; SCR frequency: 1/min; SCR amplitude: 0.1 uS. RT slopes in ms.
Bootstrap: seed 4596147 reset for each model; R Mersenne-Twister/Inversion/Rejection; quantile type 7.
BH family = 14 additive effects plus the interaction coefficient.

Baseline-HR by HR-change interaction: logit coefficient; both HR terms scaled per 10 bpm
                                    term     estimate     SE_HC3 df     CI_low   CI_high         p   q_BH_15 bootstrap_low bootstrap_high
 Delta_HR_centered_10:HR_Base_centered_10 0.0008631672 0.08282915 43 -0.1661777 0.1679041 0.9917336 0.9917336    -0.1693455      0.1371741
 converged    B
      4999 4999

All fitted coefficients; individual HC3 intervals and unadjusted p values
                          model                                term      estimate      SE_HC3 df        CI_low       CI_high            p
                 HR DT_Accuracy                         (Intercept)  2.425842e+00  0.795722496 44  8.221686e-01  4.029515e+00 3.882117e-03
                 HR DT_Accuracy                         Delta_HR_10  1.703041e-01  0.077572214 44  1.396755e-02  3.266406e-01 3.345192e-02
                 HR DT_Accuracy                     HR_Baseline_bpm  2.426155e-03  0.004187273 44 -6.012739e-03  1.086505e-02 5.652664e-01
                 HR DT_Accuracy                      DT_Stimuli_100 -1.165531e-01  0.133400017 44 -3.854031e-01  1.522970e-01 3.870195e-01
      HR DT_Omission_proportion                         (Intercept) -1.373804e+00  0.829398396 44 -3.045346e+00  2.977388e-01 1.047591e-01
      HR DT_Omission_proportion                         Delta_HR_10 -1.453072e-01  0.078499580 44 -3.035127e-01  1.289828e-02 7.088095e-02
      HR DT_Omission_proportion                     HR_Baseline_bpm -3.595999e-04  0.004994332 44 -1.042502e-02  9.705815e-03 9.429269e-01
      HR DT_Omission_proportion                      DT_Stimuli_100 -2.133896e-01  0.138554121 44 -4.926271e-01  6.584790e-02 1.306934e-01
        HR Incorrect_proportion                         (Intercept) -5.574180e+00  0.888904840 44 -7.365650e+00 -3.782710e+00 1.348572e-07
        HR Incorrect_proportion                         Delta_HR_10 -1.791565e-01  0.100533388 44 -3.817682e-01  2.345525e-02 8.164135e-02
        HR Incorrect_proportion                     HR_Baseline_bpm -5.185586e-03  0.004924624 44 -1.511051e-02  4.739341e-03 2.980935e-01
        HR Incorrect_proportion                      DT_Stimuli_100  5.500047e-01  0.176326497 44  1.946420e-01  9.053674e-01 3.195269e-03
                    HR DT_RT_ms                         (Intercept)  1.308216e+03 51.451856644 44  1.204521e+03  1.411910e+03 6.109504e-28
                    HR DT_RT_ms                         Delta_HR_10  1.055007e+01  5.174654906 44  1.212357e-01  2.097890e+01 4.750638e-02
                    HR DT_RT_ms                     HR_Baseline_bpm -1.505133e-01  0.501397849 44 -1.161014e+00  8.599877e-01 7.654480e-01
                    HR DT_RT_ms                      DT_Stimuli_100 -1.043268e+02 10.733015855 44 -1.259578e+02 -8.269586e+01 1.591429e-12
                 SC DT_Accuracy                         (Intercept)  2.582127e+00  0.807216750 44  9.552881e-01  4.208965e+00 2.559263e-03
                 SC DT_Accuracy                        SC_Change_uS  6.896408e-03  0.019379290 44 -3.215998e-02  4.595280e-02 7.236424e-01
                 SC DT_Accuracy                      SC_Baseline_uS  1.788721e-02  0.020164834 44 -2.275234e-02  5.852676e-02 3.798753e-01
                 SC DT_Accuracy                      DT_Stimuli_100 -7.030605e-02  0.144198957 44 -3.609199e-01  2.203079e-01 6.282801e-01
      SC DT_Omission_proportion                         (Intercept) -1.376370e+00  0.825820555 44 -3.040702e+00  2.879620e-01 1.026842e-01
      SC DT_Omission_proportion                        SC_Change_uS -9.709125e-03  0.024524399 44 -5.913480e-02  3.971655e-02 6.940946e-01
      SC DT_Omission_proportion                      SC_Baseline_uS -1.500288e-02  0.028332938 44 -7.210417e-02  4.209841e-02 5.991060e-01
      SC DT_Omission_proportion                      DT_Stimuli_100 -2.501683e-01  0.144680884 44 -5.417535e-01  4.141686e-02 9.080186e-02
        SC Incorrect_proportion                         (Intercept) -5.937598e+00  0.915767269 44 -7.783206e+00 -4.091991e+00 6.554571e-08
        SC Incorrect_proportion                        SC_Change_uS -1.669307e-03  0.025786503 44 -5.363859e-02  5.029997e-02 9.486777e-01
        SC Incorrect_proportion                      SC_Baseline_uS -2.209235e-02  0.026918220 44 -7.634245e-02  3.215776e-02 4.162297e-01
        SC Incorrect_proportion                      DT_Stimuli_100  4.990766e-01  0.171728566 44  1.529804e-01  8.451728e-01 5.708425e-03
                    SC DT_RT_ms                         (Intercept)  1.313244e+03 49.683875609 44  1.213112e+03  1.413375e+03 1.227785e-28
                    SC DT_RT_ms                        SC_Change_uS  2.008993e+00  2.897652580 44 -3.830842e+00  7.848828e+00 4.917541e-01
                    SC DT_RT_ms                      SC_Baseline_uS -1.161162e+00  3.600334037 44 -8.417159e+00  6.094834e+00 7.485900e-01
                    SC DT_RT_ms                      DT_Stimuli_100 -1.043382e+02  9.240612713 44 -1.229615e+02 -8.571499e+01 1.385094e-14
                SCL DT_Accuracy                         (Intercept)  2.669828e+00  0.806651546 44  1.044129e+00  4.295527e+00 1.869469e-03
                SCL DT_Accuracy                        Delta_SCL_uS  6.342254e-03  0.020934662 44 -3.584878e-02  4.853329e-02 7.633522e-01
                SCL DT_Accuracy                     Baseline_SCL_uS  1.953552e-02  0.024364214 44 -2.956732e-02  6.863837e-02 4.269709e-01
                SCL DT_Accuracy                      DT_Stimuli_100 -8.460393e-02  0.146286879 44 -3.794258e-01  2.102179e-01 5.659815e-01
     SCL DT_Omission_proportion                         (Intercept) -1.405729e+00  0.835409072 44 -3.089386e+00  2.779269e-01 9.952050e-02
     SCL DT_Omission_proportion                        Delta_SCL_uS -9.904654e-03  0.027974778 44 -6.628411e-02  4.647481e-02 7.249876e-01
     SCL DT_Omission_proportion                     Baseline_SCL_uS -2.876173e-02  0.035322609 44 -9.994977e-02  4.242631e-02 4.198821e-01
     SCL DT_Omission_proportion                      DT_Stimuli_100 -2.384040e-01  0.147758618 44 -5.361920e-01  5.938388e-02 1.137926e-01
            SCRfreq DT_Accuracy                         (Intercept)  2.719942e+00  0.773785443 44  1.160480e+00  4.279404e+00 1.031878e-03
            SCRfreq DT_Accuracy               Delta_SCR_model_per_min  4.465339e-03  0.015336199 44 -2.644274e-02  3.537342e-02 7.722946e-01
            SCRfreq DT_Accuracy            Baseline_SCR_model_per_min  1.655353e-02  0.020609200 44 -2.498158e-02  5.808865e-02 4.261706e-01
            SCRfreq DT_Accuracy                      DT_Stimuli_100 -9.660678e-02  0.145243595 44 -3.893260e-01  1.961125e-01 5.094350e-01
 SCRfreq DT_Omission_proportion                         (Intercept) -1.454083e+00  0.814789585 44 -3.096183e+00  1.880177e-01 8.122023e-02
 SCRfreq DT_Omission_proportion               Delta_SCR_model_per_min -9.193664e-03  0.015930769 44 -4.130002e-02  2.291269e-02 5.668124e-01
 SCRfreq DT_Omission_proportion            Baseline_SCR_model_per_min -1.258519e-02  0.021919868 44 -5.676178e-02  3.159140e-02 5.687931e-01
 SCRfreq DT_Omission_proportion                      DT_Stimuli_100 -2.328736e-01  0.145107606 44 -5.253188e-01  5.957155e-02 1.156850e-01
             SCRamp DT_Accuracy                         (Intercept)  3.067571e+00  0.951780177 44  1.149385e+00  4.985758e+00 2.391020e-03
```

SCRamp	DT_Accuracy	Delta_SCR_amplitude_zero_coded_uS	-1.238685e+00	1.361216212	44	-3.982036e+00	1.504666e+00	3.677892e-01
SCRamp	DT_Accuracy	Baseline_SCR_amplitude_zero_coded_uS	-8.744515e-01	0.826972627	44	-2.541105e+00	7.922023e-01	2.960943e-01
SCRamp	DT_Accuracy	DT_Stimuli_100	-9.773787e-02	0.148550521	44	-3.971218e-01	2.016460e-01	5.140021e-01
SCRamp	DT_Omission_proportion	(Intercept)	-1.689638e+00	0.827288484	44	-3.356928e+00	-2.234763e-02	4.713458e-02
SCRamp	DT_Omission_proportion	Delta_SCR_amplitude_zero_coded_uS	3.998434e-01	0.908404936	44	-1.430926e+00	2.230613e+00	6.619760e-01
SCRamp	DT_Omission_proportion	Baseline_SCR_amplitude_zero_coded_uS	4.169662e-01	0.847096874	44	-1.290245e+00	2.124178e+00	6.250042e-01
SCRamp	DT_Omission_proportion	DT_Stimuli_100	-2.301558e-01	0.143062736	44	-5.184798e-01	5.816821e-02	1.148182e-01
HR	interaction	(Intercept)	2.950135e+00	0.758187282	43	1.421105e+00	4.479165e+00	3.420032e-04
HR	interaction	Delta_HR_centered_10	1.704990e-01	0.079583922	43	1.000272e-02	3.309953e-01	3.786673e-02
HR	interaction	HR_Base_centered_10	2.428374e-02	0.047489586	43	-7.148813e-02	1.200556e-01	6.117227e-01
HR	interaction	DT_Stimuli_100	-1.165417e-01	0.134063041	43	-3.869056e-01	1.538222e-01	3.895068e-01
HR	interaction	Delta_HR_centered_10:HR_Base_centered_10	8.631672e-04	0.082829148	43	-1.661777e-01	1.679041e-01	9.917336e-01

04 | Table S2 Spearman, Theil-Sen and leave-one-out

Table S2: Spearman, Pearson leave-one-out and Theil-Sen

change	outcome	rho	p	LOO_r_low	LOO_r_high	Theil_Sen	bootstrap_low	bootstrap_high	B	q_BH_10
HR	Accuracy_pct	0.1924172932	0.1901117	0.2856913915	0.37789001	0.75548245	-0.4985955	2.2012164	1999	0.8260442
HR	DT_Omitted	-0.2149183188	0.1423891	-0.3289809158	-0.23785470	-3.79772626	-9.1120709	0.9055161	1999	0.8260442
HR	DT_Incorrect	-0.0813344205	0.5826215	-0.2493481919	-0.12503479	-0.94483407	-5.7033955	4.3495885	1999	0.8260442
HR	DT_Correct	0.1314532335	0.3731431	0.1018266852	0.23150892	9.58206735	-9.8286491	26.8994922	1999	0.8260442
HR	DT_RT_ms	0.0804472914	0.5867541	0.0457663480	0.13042638	5.78844730	-18.8757905	33.5714939	1999	0.8260442
SC	Accuracy_pct	0.0324042663	0.8269283	0.0098605415	0.13433679	0.01615934	-0.3370908	0.3577191	1999	0.9188092
SC	DT_Omitted	-0.0649753057	0.6608353	-0.1407332288	-0.05254251	-0.30788177	-1.8578262	1.4529604	1999	0.8260442
SC	DT_Incorrect	0.0708484197	0.6322833	-0.0873434954	0.07000966	0.18908414	-1.2975658	1.4532963	1999	0.8260442
SC	DT_Correct	-0.0001900005	0.9989774	-0.0204574766	0.08780255	0.00000000	-4.9934494	5.3626586	1999	0.9989774
SC	DT_RT_ms	0.1186624690	0.4218066	0.0005776065	0.14821923	2.74240940	-3.8812015	10.8714217	1999	0.8260442

Spearman p: two-sided t approximation (exact=FALSE); BH family of 10.
Theil-Sen: median of finite pairwise slopes; 1999 paired-participant resamples.
Seed 4596147 reset for each slope; percentile interval, quantile type 7.

Pearson leave-one-out results for every omitted participant

change	outcome	omitted_ID	r
HR	Accuracy_pct	S001	0.3395752778
HR	Accuracy_pct	S002	0.3421626744
HR	Accuracy_pct	S003	0.3271285834
HR	Accuracy_pct	S004	0.3441040477
HR	Accuracy_pct	S005	0.3536467654
HR	Accuracy_pct	S006	0.3406802710
HR	Accuracy_pct	S007	0.2856913915
HR	Accuracy_pct	S008	0.3313663456
HR	Accuracy_pct	S009	0.2952601837
HR	Accuracy_pct	S010	0.3292936227
HR	Accuracy_pct	S011	0.3272903609
HR	Accuracy_pct	S012	0.3549716517
HR	Accuracy_pct	S013	0.3400307778
HR	Accuracy_pct	S014	0.3492328994
HR	Accuracy_pct	S015	0.3359475123
HR	Accuracy_pct	S016	0.3326959203
HR	Accuracy_pct	S017	0.3301178958
HR	Accuracy_pct	S018	0.2963723219
HR	Accuracy_pct	S019	0.3367263589
HR	Accuracy_pct	S020	0.3391314485
HR	Accuracy_pct	S021	0.3550411860
HR	Accuracy_pct	S022	0.2941611487
HR	Accuracy_pct	S023	0.3504381883
HR	Accuracy_pct	S024	0.3381729742
HR	Accuracy_pct	S025	0.3366413465
HR	Accuracy_pct	S026	0.3444017668
HR	Accuracy_pct	S027	0.3349649897
HR	Accuracy_pct	S028	0.3394083077
HR	Accuracy_pct	S029	0.3635430425
HR	Accuracy_pct	S030	0.3359817868
HR	Accuracy_pct	S031	0.2894923455
HR	Accuracy_pct	S032	0.3423629706
HR	Accuracy_pct	S033	0.3400078494
HR	Accuracy_pct	S034	0.3573605492
HR	Accuracy_pct	S035	0.3567404502
HR	Accuracy_pct	S036	0.3435493812
HR	Accuracy_pct	S037	0.3513203991
HR	Accuracy_pct	S038	0.3620351903
HR	Accuracy_pct	S039	0.3375373791
HR	Accuracy_pct	S040	0.3343963447
HR	Accuracy_pct	S041	0.3368166107
HR	Accuracy_pct	S042	0.3398063996
HR	Accuracy_pct	S043	0.3376864006
HR	Accuracy_pct	S044	0.3421383099
HR	Accuracy_pct	S045	0.3579369763
HR	Accuracy_pct	S046	0.3778900140
HR	Accuracy_pct	S047	0.3469271225
HR	Accuracy_pct	S048	0.3413456341
HR	DT_Omitted	S001	-0.2920802336
HR	DT_Omitted	S002	-0.2913449739
HR	DT_Omitted	S003	-0.2816193600
HR	DT_Omitted	S004	-0.3086823590
HR	DT_Omitted	S005	-0.3180581686
HR	DT_Omitted	S006	-0.2906245582
HR	DT_Omitted	S007	-0.2378547002
HR	DT_Omitted	S008	-0.2848221899
HR	DT_Omitted	S009	-0.2625558203
HR	DT_Omitted	S010	-0.2814009364
HR	DT_Omitted	S011	-0.2740119753
HR	DT_Omitted	S012	-0.3117882006
HR	DT_Omitted	S013	-0.2981681687
HR	DT_Omitted	S014	-0.2778403022
HR	DT_Omitted	S015	-0.2886904610
HR	DT_Omitted	S016	-0.3063053482
HR	DT_Omitted	S017	-0.2946579441
HR	DT_Omitted	S018	-0.2423917466
HR	DT_Omitted	S019	-0.2891820973
HR	DT_Omitted	S020	-0.2946441070
HR	DT_Omitted	S021	-0.3289809158
HR	DT_Omitted	S022	-0.2602238216

HR	DT_Omitted	S023	-0.2971104567
HR	DT_Omitted	S024	-0.2910454194
HR	DT_Omitted	S025	-0.2824671568
HR	DT_Omitted	S026	-0.2895652662
HR	DT_Omitted	S027	-0.2864051128
HR	DT_Omitted	S028	-0.2871821678
HR	DT_Omitted	S029	-0.2970763366
HR	DT_Omitted	S030	-0.2877574274
HR	DT_Omitted	S031	-0.2438034487
HR	DT_Omitted	S032	-0.2964248078
HR	DT_Omitted	S033	-0.2802917617
HR	DT_Omitted	S034	-0.3058124956
HR	DT_Omitted	S035	-0.3076695502
HR	DT_Omitted	S036	-0.2959907121
HR	DT_Omitted	S037	-0.2996477122
HR	DT_Omitted	S038	-0.3116488728
HR	DT_Omitted	S039	-0.2904996379
HR	DT_Omitted	S040	-0.2845014635
HR	DT_Omitted	S041	-0.2845917003
HR	DT_Omitted	S042	-0.2945395455
HR	DT_Omitted	S043	-0.2922281722
HR	DT_Omitted	S044	-0.2959210916
HR	DT_Omitted	S045	-0.3066975680
HR	DT_Omitted	S046	-0.3168607064
HR	DT_Omitted	S047	-0.3114403964
HR	DT_Omitted	S048	-0.2976016189
HR	DT_Incorrect	S001	-0.2124203996
HR	DT_Incorrect	S002	-0.2108107054
HR	DT_Incorrect	S003	-0.1847526076
HR	DT_Incorrect	S004	-0.2072560170
HR	DT_Incorrect	S005	-0.2127052780
HR	DT_Incorrect	S006	-0.2129793543
HR	DT_Incorrect	S007	-0.1788253860
HR	DT_Incorrect	S008	-0.2070729345
HR	DT_Incorrect	S009	-0.1250347918
HR	DT_Incorrect	S010	-0.2118588586
HR	DT_Incorrect	S011	-0.2089227802
HR	DT_Incorrect	S012	-0.2252764910
HR	DT_Incorrect	S013	-0.2079929288
HR	DT_Incorrect	S014	-0.2493481919
HR	DT_Incorrect	S015	-0.2045154655
HR	DT_Incorrect	S016	-0.1898120871
HR	DT_Incorrect	S017	-0.1864951518
HR	DT_Incorrect	S018	-0.1874902440
HR	DT_Incorrect	S019	-0.2079502948
HR	DT_Incorrect	S020	-0.2071038122
HR	DT_Incorrect	S021	-0.2038646951
HR	DT_Incorrect	S022	-0.1889425450
HR	DT_Incorrect	S023	-0.2086372460
HR	DT_Incorrect	S024	-0.2104184922
HR	DT_Incorrect	S025	-0.2242617249
HR	DT_Incorrect	S026	-0.2241369635
HR	DT_Incorrect	S027	-0.2071067772
HR	DT_Incorrect	S028	-0.2192245779
HR	DT_Incorrect	S029	-0.2276569986
HR	DT_Incorrect	S030	-0.2082819826
HR	DT_Incorrect	S031	-0.1737572276
HR	DT_Incorrect	S032	-0.2094840912
HR	DT_Incorrect	S033	-0.2216768281
HR	DT_Incorrect	S034	-0.2204191342
HR	DT_Incorrect	S035	-0.2176495171
HR	DT_Incorrect	S036	-0.2287637864
HR	DT_Incorrect	S037	-0.2187870835
HR	DT_Incorrect	S038	-0.2086440391
HR	DT_Incorrect	S039	-0.2140004714
HR	DT_Incorrect	S040	-0.2189251567
HR	DT_Incorrect	S041	-0.2219119350
HR	DT_Incorrect	S042	-0.2096606116
HR	DT_Incorrect	S043	-0.2080040084
HR	DT_Incorrect	S044	-0.2109498217
HR	DT_Incorrect	S045	-0.2230954038
HR	DT_Incorrect	S046	-0.2436380920
HR	DT_Incorrect	S047	-0.2043503681
HR	DT_Incorrect	S048	-0.2096542865
HR	DT_Correct	S001	0.1801853550
HR	DT_Correct	S002	0.1807420404
HR	DT_Correct	S003	0.2315089199
HR	DT_Correct	S004	0.1916919599
HR	DT_Correct	S005	0.1721241114
HR	DT_Correct	S006	0.1810515338
HR	DT_Correct	S007	0.1751913645
HR	DT_Correct	S008	0.1716227567
HR	DT_Correct	S009	0.2014482856
HR	DT_Correct	S010	0.1634832136
HR	DT_Correct	S011	0.1864745828
HR	DT_Correct	S012	0.1631968668
HR	DT_Correct	S013	0.1772766662
HR	DT_Correct	S014	0.1562170055
HR	DT_Correct	S015	0.1978186920
HR	DT_Correct	S016	0.1767220332
HR	DT_Correct	S017	0.2070050828
HR	DT_Correct	S018	0.1944442834
HR	DT_Correct	S019	0.1791028686
HR	DT_Correct	S020	0.1941053810
HR	DT_Correct	S021	0.1899170028
HR	DT_Correct	S022	0.1018266852
HR	DT_Correct	S023	0.2159505820
HR	DT_Correct	S024	0.1790817129
HR	DT_Correct	S025	0.1660367629
HR	DT_Correct	S026	0.1708388064
HR	DT_Correct	S027	0.1890227529
HR	DT_Correct	S028	0.1759071048
HR	DT_Correct	S029	0.1926305406
HR	DT_Correct	S030	0.1821566914
HR	DT_Correct	S031	0.1624857409

HR	DT_Correct	S032	0.1831829912
HR	DT_Correct	S033	0.1888732317
HR	DT_Correct	S034	0.1898824540
HR	DT_Correct	S035	0.1938547262
HR	DT_Correct	S036	0.1431129058
HR	DT_Correct	S037	0.1830281745
HR	DT_Correct	S038	0.2102078331
HR	DT_Correct	S039	0.1791780054
HR	DT_Correct	S040	0.1712561705
HR	DT_Correct	S041	0.1659663822
HR	DT_Correct	S042	0.1786761377
HR	DT_Correct	S043	0.1799326209
HR	DT_Correct	S044	0.1788805072
HR	DT_Correct	S045	0.1780242284
HR	DT_Correct	S046	0.1657755968
HR	DT_Correct	S047	0.1870544077
HR	DT_Correct	S048	0.1793113420
HR	DT_RT_ms	S001	0.0982268958
HR	DT_RT_ms	S002	0.0917341775
HR	DT_RT_ms	S003	0.0507449815
HR	DT_RT_ms	S004	0.0940849293
HR	DT_RT_ms	S005	0.0784847965
HR	DT_RT_ms	S006	0.0920861819
HR	DT_RT_ms	S007	0.0457663480
HR	DT_RT_ms	S008	0.0929801088
HR	DT_RT_ms	S009	0.0560510690
HR	DT_RT_ms	S010	0.1148711305
HR	DT_RT_ms	S011	0.0788968457
HR	DT_RT_ms	S012	0.1234283088
HR	DT_RT_ms	S013	0.0830771686
HR	DT_RT_ms	S014	0.1296172851
HR	DT_RT_ms	S015	0.0823004718
HR	DT_RT_ms	S016	0.1304263783
HR	DT_RT_ms	S017	0.0648226817
HR	DT_RT_ms	S018	0.0462154816
HR	DT_RT_ms	S019	0.0912157662
HR	DT_RT_ms	S020	0.0884265890
HR	DT_RT_ms	S021	0.0939608408
HR	DT_RT_ms	S022	0.1273907140
HR	DT_RT_ms	S023	0.0652776017
HR	DT_RT_ms	S024	0.0986321036
HR	DT_RT_ms	S025	0.1057657769
HR	DT_RT_ms	S026	0.1126394025
HR	DT_RT_ms	S027	0.0800815812
HR	DT_RT_ms	S028	0.0903400256
HR	DT_RT_ms	S029	0.0863981773
HR	DT_RT_ms	S030	0.0890855001
HR	DT_RT_ms	S031	0.0634702475
HR	DT_RT_ms	S032	0.0917963298
HR	DT_RT_ms	S033	0.0788102395
HR	DT_RT_ms	S034	0.1100293915
HR	DT_RT_ms	S035	0.0944659213
HR	DT_RT_ms	S036	0.1240662257
HR	DT_RT_ms	S037	0.0936235832
HR	DT_RT_ms	S038	0.0834747010
HR	DT_RT_ms	S039	0.0939762494
HR	DT_RT_ms	S040	0.0990239258
HR	DT_RT_ms	S041	0.1114837445
HR	DT_RT_ms	S042	0.0920113649
HR	DT_RT_ms	S043	0.0950501702
HR	DT_RT_ms	S044	0.0977864345
HR	DT_RT_ms	S045	0.1058868962
HR	DT_RT_ms	S046	0.1153345087
HR	DT_RT_ms	S047	0.0921848291
HR	DT_RT_ms	S048	0.0955464338
SC	Accuracy_pct	S001	0.0968246012
SC	Accuracy_pct	S002	0.0482157599
SC	Accuracy_pct	S003	0.0708546558
SC	Accuracy_pct	S004	0.0646438907
SC	Accuracy_pct	S005	0.0952912729
SC	Accuracy_pct	S006	0.0925714976
SC	Accuracy_pct	S007	0.0434954198
SC	Accuracy_pct	S008	0.0748918519
SC	Accuracy_pct	S009	0.0098605415
SC	Accuracy_pct	S010	0.0821645712
SC	Accuracy_pct	S011	0.0841654692
SC	Accuracy_pct	S012	0.0875512040
SC	Accuracy_pct	S013	0.0791779022
SC	Accuracy_pct	S014	0.0772238248
SC	Accuracy_pct	S015	0.0751219020
SC	Accuracy_pct	S016	0.0919471129
SC	Accuracy_pct	S017	0.0678382926
SC	Accuracy_pct	S018	0.1222500948
SC	Accuracy_pct	S019	0.0788250966
SC	Accuracy_pct	S020	0.0834100622
SC	Accuracy_pct	S021	0.0589758527
SC	Accuracy_pct	S022	0.0685678343
SC	Accuracy_pct	S023	0.0895551806
SC	Accuracy_pct	S024	0.0784401776
SC	Accuracy_pct	S025	0.0740411871
SC	Accuracy_pct	S026	0.0796739842
SC	Accuracy_pct	S027	0.0535919575
SC	Accuracy_pct	S028	0.0797284137
SC	Accuracy_pct	S029	0.0610553338
SC	Accuracy_pct	S030	0.0742649423
SC	Accuracy_pct	S031	0.1343367936
SC	Accuracy_pct	S032	0.0561957281
SC	Accuracy_pct	S033	0.0798081150
SC	Accuracy_pct	S034	0.0997639596
SC	Accuracy_pct	S035	0.0736855014
SC	Accuracy_pct	S036	0.0789381152
SC	Accuracy_pct	S037	0.0931372355
SC	Accuracy_pct	S038	0.0746301989
SC	Accuracy_pct	S039	0.0798453020
SC	Accuracy_pct	S040	0.0909068895

SC	Accuracy_pct	S041	0.0769995487
SC	Accuracy_pct	S042	0.0789201564
SC	Accuracy_pct	S043	0.0805973617
SC	Accuracy_pct	S044	0.0785502900
SC	Accuracy_pct	S045	0.0672252010
SC	Accuracy_pct	S046	0.1206721473
SC	Accuracy_pct	S047	0.0663153187
SC	Accuracy_pct	S048	0.0780328574
SC	DT_Omitted	S001	-0.1107379664
SC	DT_Omitted	S002	-0.0764087233
SC	DT_Omitted	S003	-0.0860164661
SC	DT_Omitted	S004	-0.0621840791
SC	DT_Omitted	S005	-0.1216447019
SC	DT_Omitted	S006	-0.0901550505
SC	DT_Omitted	S007	-0.0596094436
SC	DT_Omitted	S008	-0.0892618189
SC	DT_Omitted	S009	-0.0635110586
SC	DT_Omitted	S010	-0.0958560440
SC	DT_Omitted	S011	-0.1052989181
SC	DT_Omitted	S012	-0.1042662202
SC	DT_Omitted	S013	-0.0739272935
SC	DT_Omitted	S014	-0.1141287662
SC	DT_Omitted	S015	-0.0892718555
SC	DT_Omitted	S016	-0.0900227810
SC	DT_Omitted	S017	-0.0920051311
SC	DT_Omitted	S018	-0.1407332288
SC	DT_Omitted	S019	-0.0924744126
SC	DT_Omitted	S020	-0.1086553428
SC	DT_Omitted	S021	-0.0525425146
SC	DT_Omitted	S022	-0.0844686913
SC	DT_Omitted	S023	-0.0975641895
SC	DT_Omitted	S024	-0.0917833770
SC	DT_Omitted	S025	-0.0721324167
SC	DT_Omitted	S026	-0.0893689096
SC	DT_Omitted	S027	-0.0547208667
SC	DT_Omitted	S028	-0.0837218727
SC	DT_Omitted	S029	-0.0864981707
SC	DT_Omitted	S030	-0.0867040315
SC	DT_Omitted	S031	-0.1398883585
SC	DT_Omitted	S032	-0.0641922923
SC	DT_Omitted	S033	-0.1184234752
SC	DT_Omitted	S034	-0.1099035357
SC	DT_Omitted	S035	-0.0877272200
SC	DT_Omitted	S036	-0.0929656828
SC	DT_Omitted	S037	-0.1025407779
SC	DT_Omitted	S038	-0.0887996477
SC	DT_Omitted	S039	-0.0979795751
SC	DT_Omitted	S040	-0.1258864692
SC	DT_Omitted	S041	-0.0883802716
SC	DT_Omitted	S042	-0.0946185513
SC	DT_Omitted	S043	-0.0917355005
SC	DT_Omitted	S044	-0.0917690271
SC	DT_Omitted	S045	-0.0822201676
SC	DT_Omitted	S046	-0.1210416500
SC	DT_Omitted	S047	-0.0595535663
SC	DT_Omitted	S048	-0.0929042612
SC	DT_Incorrect	S001	-0.0822030226
SC	DT_Incorrect	S002	-0.0187595164
SC	DT_Incorrect	S003	-0.0349135885
SC	DT_Incorrect	S004	-0.0615031579
SC	DT_Incorrect	S005	-0.0518168657
SC	DT_Incorrect	S006	-0.0691512139
SC	DT_Incorrect	S007	-0.0286851117
SC	DT_Incorrect	S008	-0.0467760409
SC	DT_Incorrect	S009	0.0700096627
SC	DT_Incorrect	S010	-0.0475532349
SC	DT_Incorrect	S011	-0.0482972627
SC	DT_Incorrect	S012	-0.0574465477
SC	DT_Incorrect	S013	-0.0582899208
SC	DT_Incorrect	S014	-0.0190370563
SC	DT_Incorrect	S015	-0.0415610167
SC	DT_Incorrect	S016	-0.0676793652
SC	DT_Incorrect	S017	-0.0268651969
SC	DT_Incorrect	S018	-0.0669541947
SC	DT_Incorrect	S019	-0.0480609910
SC	DT_Incorrect	S020	-0.0292245606
SC	DT_Incorrect	S021	-0.0601915984
SC	DT_Incorrect	S022	-0.0423607909
SC	DT_Incorrect	S023	-0.0457788575
SC	DT_Incorrect	S024	-0.0466358611
SC	DT_Incorrect	S025	-0.0698590286
SC	DT_Incorrect	S026	-0.0550872618
SC	DT_Incorrect	S027	-0.0357583713
SC	DT_Incorrect	S028	-0.0613994059
SC	DT_Incorrect	S029	-0.0319821044
SC	DT_Incorrect	S030	-0.0462984600
SC	DT_Incorrect	S031	-0.0767541295
SC	DT_Incorrect	S032	-0.0453386197
SC	DT_Incorrect	S033	-0.0322516902
SC	DT_Incorrect	S034	-0.0602346410
SC	DT_Incorrect	S035	-0.0457923949
SC	DT_Incorrect	S036	-0.0569243137
SC	DT_Incorrect	S037	-0.0595298098
SC	DT_Incorrect	S038	-0.0483402893
SC	DT_Incorrect	S039	-0.0441030600
SC	DT_Incorrect	S040	-0.0334521216
SC	DT_Incorrect	S041	-0.0531080620
SC	DT_Incorrect	S042	-0.0477670682
SC	DT_Incorrect	S043	-0.0505048474
SC	DT_Incorrect	S044	-0.0482715963
SC	DT_Incorrect	S045	-0.0373343614
SC	DT_Incorrect	S046	-0.0873434954
SC	DT_Incorrect	S047	-0.0641023376
SC	DT_Incorrect	S048	-0.0473246662
SC	DT_Correct	S001	-0.0204574766

```
SC   DT_Correct       S002  0.0442731371
SC   DT_Correct       S003  0.0623149558
SC   DT_Correct       S004  0.0151047611
SC   DT_Correct       S005  0.0278499433
SC   DT_Correct       S006  0.0461313840
SC   DT_Correct       S007  0.0356209179
SC   DT_Correct       S008  0.0379835001
SC   DT_Correct       S009  0.0559083710
SC   DT_Correct       S010  0.0480841503
SC   DT_Correct       S011  0.0406169208
SC   DT_Correct       S012  0.0277323795
SC   DT_Correct       S013  0.0608391563
SC   DT_Correct       S014  0.0714512963
SC   DT_Correct       S015  0.0549898793
SC   DT_Correct       S016  0.0495418382
SC   DT_Correct       S017  0.0593331920
SC   DT_Correct       S018  0.0366251561
SC   DT_Correct       S019  0.0420306890
SC   DT_Correct       S020  0.0878025545
SC   DT_Correct       S021  0.0293155074
SC   DT_Correct       S022  0.0308695485
SC   DT_Correct       S023  0.0772488362
SC   DT_Correct       S024  0.0469633536
SC   DT_Correct       S025  0.0078731950
SC   DT_Correct       S026  0.0346325493
SC   DT_Correct       S027  0.0785982580
SC   DT_Correct       S028  0.0320987839
SC   DT_Correct       S029  0.0303328621
SC   DT_Correct       S030  0.0435030787
SC   DT_Correct       S031  0.0565522392
SC   DT_Correct       S032  0.0207317470
SC   DT_Correct       S033  0.0315666819
SC   DT_Correct       S034  0.0518858596
SC   DT_Correct       S035  0.0379960835
SC   DT_Correct       S036  0.0176728101
SC   DT_Correct       S037  0.0443637860
SC   DT_Correct       S038  0.0357520094
SC   DT_Correct       S039  0.0480329975
SC   DT_Correct       S040  0.0714190139
SC   DT_Correct       S041  0.0336732997
SC   DT_Correct       S042  0.0395643661
SC   DT_Correct       S043  0.0436036357
SC   DT_Correct       S044  0.0430665697
SC   DT_Correct       S045  0.0459536686
SC   DT_Correct       S046  0.0184673867
SC   DT_Correct       S047  0.0312195013
SC   DT_Correct       S048  0.0408791266
SC     DT_RT_ms       S001  0.1482192302
SC     DT_RT_ms       S002  0.0567545180
SC     DT_RT_ms       S003  0.0604801892
SC     DT_RT_ms       S004  0.0713832136
SC     DT_RT_ms       S005  0.0586017434
SC     DT_RT_ms       S006  0.0791280496
SC     DT_RT_ms       S007  0.0552310656
SC     DT_RT_ms       S008  0.0783805809
SC     DT_RT_ms       S009  0.0496138627
SC     DT_RT_ms       S010  0.0757891017
SC     DT_RT_ms       S011  0.0828675406
SC     DT_RT_ms       S012  0.0998914076
SC     DT_RT_ms       S013  0.1228828109
SC     DT_RT_ms       S014  0.0489465243
SC     DT_RT_ms       S015  0.0680019528
SC     DT_RT_ms       S016  0.0570652980
SC     DT_RT_ms       S017  0.0569785076
SC     DT_RT_ms       S018  0.1126610081
SC     DT_RT_ms       S019  0.0782223746
SC     DT_RT_ms       S020  0.0540976662
SC     DT_RT_ms       S021  0.0766824515
SC     DT_RT_ms       S022  0.0804616266
SC     DT_RT_ms       S023  0.0459906749
SC     DT_RT_ms       S024  0.0731221087
SC     DT_RT_ms       S025  0.1012296200
SC     DT_RT_ms       S026  0.0900758772
SC     DT_RT_ms       S027  0.0005776065
SC     DT_RT_ms       S028  0.0752989889
SC     DT_RT_ms       S029  0.0864792473
SC     DT_RT_ms       S030  0.0742007477
SC     DT_RT_ms       S031  0.0989643739
SC     DT_RT_ms       S032  0.0849306564
SC     DT_RT_ms       S033  0.1037044075
SC     DT_RT_ms       S034  0.1082761521
SC     DT_RT_ms       S035  0.0777394257
SC     DT_RT_ms       S036  0.0957752711
SC     DT_RT_ms       S037  0.0804184024
SC     DT_RT_ms       S038  0.0855800074
SC     DT_RT_ms       S039  0.0760627559
SC     DT_RT_ms       S040  0.0667052099
SC     DT_RT_ms       S041  0.0877192070
SC     DT_RT_ms       S042  0.0780124563
SC     DT_RT_ms       S043  0.0752945840
SC     DT_RT_ms       S044  0.0770384526
SC     DT_RT_ms       S045  0.0667366054
SC     DT_RT_ms       S046  0.1087763420
SC     DT_RT_ms       S047  0.0812803967
SC     DT_RT_ms       S048  0.0788390060
```

05 | Table S3 sensitivity and influence diagnostics

```
Auxiliary covariates: Female, KSS_1_9 and Sleep_Duration_h from original OMV, joined by Subject_ID.

Table S3: HR-accuracy specification sensitivity
              specification  n AME_pp_per_10bpm   logit_b      HC3_low  HC3_high
        Main adjusted model 48         1.419034 0.1703041  0.013967546 0.3266406
            Add sex and KSS 48         1.396167 0.1676285  0.011817453 0.3234396
```

Add sex and sleep duration	48	1.370078	0.1644900	0.013933433	0.3150466
Omit S004	47	1.441755	0.1722004	0.016342518	0.3280582
Omit S009	47	1.053579	0.1292367	-0.012443629	0.2709171
Omit S042	47	1.437695	0.1725066	0.014735203	0.3302780
Omit S011, S013, S042	45	1.378013	0.1665292	0.004110817	0.3289475
Replace stimuli by total	48	1.407500	0.1690019	0.021391944	0.3166118

AME before adding DT_Stimuli: 1.371078 pp per 10 bpm

All participant deletions: adjusted HR-accuracy AME

omitted_ID	AME
S001	1.417590
S002	1.423839
S003	1.420547
S004	1.441755
S005	1.455063
S006	1.424538
S007	1.214750
S008	1.376846
S009	1.053579
S010	1.357482
S011	1.353820
S012	1.471375
S013	1.426636
S014	1.485752
S015	1.410937
S016	1.454411
S017	1.431659
S018	1.234528
S019	1.414524
S020	1.427134
S021	1.496469
S022	1.344738
S023	1.510890
S024	1.421891
S025	1.414507
S026	1.441412
S027	1.404721
S028	1.419861
S029	1.514315
S030	1.408311
S031	1.152587
S032	1.433014
S033	1.441086
S034	1.522783
S035	1.517108
S036	1.441808
S037	1.467409
S038	1.492671
S039	1.423020
S040	1.397929
S041	1.409404
S042	1.437695
S043	1.416472
S044	1.470098
S045	1.478410
S046	1.603389
S047	1.476986
S048	1.439147

Adjusted fractional-logit HR-accuracy diagnostics

Subject_ID	fitted	response_residual	Pearson_residual	leverage	Cook
S001	0.9029394	-0.004477826	-0.015125729	0.08849804	5.711967e-04
S002	0.9082270	0.037128198	0.128602434	0.05222130	2.253548e-02
S003	0.8849502	0.001785988	0.005597268	0.17750897	1.926836e-04
S004	0.9037767	0.027969328	0.094844325	0.03052647	6.847972e-03
S005	0.9080311	0.026584271	0.091992820	0.07048819	1.618263e-02
S006	0.9069791	0.019594372	0.067459381	0.02157085	2.403410e-03
S007	0.9273141	0.033259366	0.128107773	0.13256524	6.777064e-02
S008	0.9005684	-0.017570212	-0.058715991	0.08304858	7.981545e-03
S009	0.8883669	-0.087205826	-0.276919075	0.10255649	2.288702e-01
S010	0.9041710	-0.027943106	-0.094929441	0.09732914	2.523019e-02
S011	0.8965675	-0.023603318	-0.077509158	0.06804597	1.103200e-02
S012	0.9112924	-0.024963692	-0.087801021	0.08321627	1.788991e-02
S013	0.9041896	0.005882322	0.019985397	0.02982210	2.966167e-04
S014	0.8950250	0.015790890	0.051516479	0.08981243	6.743753e-03
S015	0.9012678	-0.007007935	-0.023492738	0.17167175	3.236650e-03
S016	0.9271704	-0.004890642	-0.018820546	0.09845370	1.005669e-03
S017	0.8872637	0.004110053	0.012995387	0.10983900	5.486969e-04
S018	0.9281054	0.035977770	0.139279588	0.09041363	4.968847e-02
S019	0.9070082	0.020826895	0.071712770	0.06540607	9.026091e-03
S020	0.9011989	0.011511648	0.038578587	0.05871469	2.311700e-03
S021	0.9167000	-0.035494339	-0.128446718	0.03375219	1.397995e-02
S022	0.9381985	0.006336589	0.026315316	0.23315662	6.435558e-03
S023	0.9226086	-0.029328002	-0.109755796	0.08102585	2.708985e-02
S024	0.9135217	-0.006324742	-0.022502451	0.08397804	1.187813e-03
S025	0.9125352	0.002849441	0.010085981	0.08923318	2.564974e-04
S026	0.9018620	0.008558459	0.028767820	0.12797266	3.264416e-03
S027	0.9177863	0.011196409	0.040760162	0.05793848	2.542231e-03
S028	0.9119420	-0.001327477	-0.004684455	0.15299413	1.096869e-04
S029	0.9169700	-0.055459240	-0.200992061	0.04058747	4.175165e-02
S030	0.9016362	-0.006543122	-0.021971095	0.03492720	4.243084e-04
S031	0.9237922	0.060278563	0.227183151	0.06659206	9.246265e-02
S032	0.9041490	0.030524339	0.103687880	0.03715456	1.009926e-02
S033	0.8970628	0.010655337	0.035064654	0.04779922	1.519279e-03
S034	0.9205961	-0.021641413	-0.080044257	0.17372410	3.821245e-02
S035	0.8948397	0.031906425	0.104011101	0.05017244	1.410164e-02
S036	0.9020351	0.006867628	0.023102539	0.13675385	2.295748e-03
S037	0.9020662	0.027387026	0.092142384	0.03151821	6.687013e-03
S038	0.9173128	-0.061050013	-0.221670551	0.11686146	1.725696e-01
S039	0.9131527	-0.014297732	-0.050771183	0.07940873	5.661138e-03
S040	0.9090845	0.018588491	0.064658106	0.05519067	6.058399e-03
S041	0.9074023	-0.006021640	-0.020773744	0.06683979	7.764023e-04
S042	0.8998868	0.009527098	0.031741025	0.08319600	2.337362e-03
S043	0.9051228	-0.001276615	-0.004356374	0.04485313	2.186942e-05

```
       S044 0.9214747      -0.012383819     -0.046037119 0.06117378 3.447829e-03
       S045 0.9099331      -0.048028304     -0.167768276 0.08240728 6.456839e-02
       S046 0.8959207       0.046386943      0.151907381 0.09919646 6.611932e-02
       S047 0.9181056      -0.022345920     -0.081493862 0.04251660 7.219056e-03
       S048 0.8989891       0.007701085      0.025555861 0.06736699 1.185608e-03

Design-matrix VIF: additive accuracy models
  change                          predictor      VIF
      HR                        Delta_HR_10 1.026042
      HR                    HR_Baseline_bpm 1.072861
      HR                     DT_Stimuli_100 1.066370
      SC                       SC_Change_uS 1.054699
      SC                     SC_Baseline_uS 1.066031
      SC                     DT_Stimuli_100 1.011488
     SCL                       Delta_SCL_uS 1.184028
     SCL                    Baseline_SCL_uS 1.182448
     SCL                     DT_Stimuli_100 1.001455
 SCRfreq             Delta_SCR_model_per_min 1.052573
 SCRfreq          Baseline_SCR_model_per_min 1.067172
 SCRfreq                     DT_Stimuli_100 1.026524
  SCRamp    Delta_SCR_amplitude_zero_coded_uS 1.509462
  SCRamp Baseline_SCR_amplitude_zero_coded_uS 1.527038
  SCRamp                     DT_Stimuli_100 1.016843

Change + baseline versus task + baseline parameterizations
  change max_fitted_difference
      HR          3.330669e-16
      SC          2.220446e-16
     SCL          2.220446e-16
 SCRfreq          1.110223e-16
  SCRamp          1.110223e-16
```

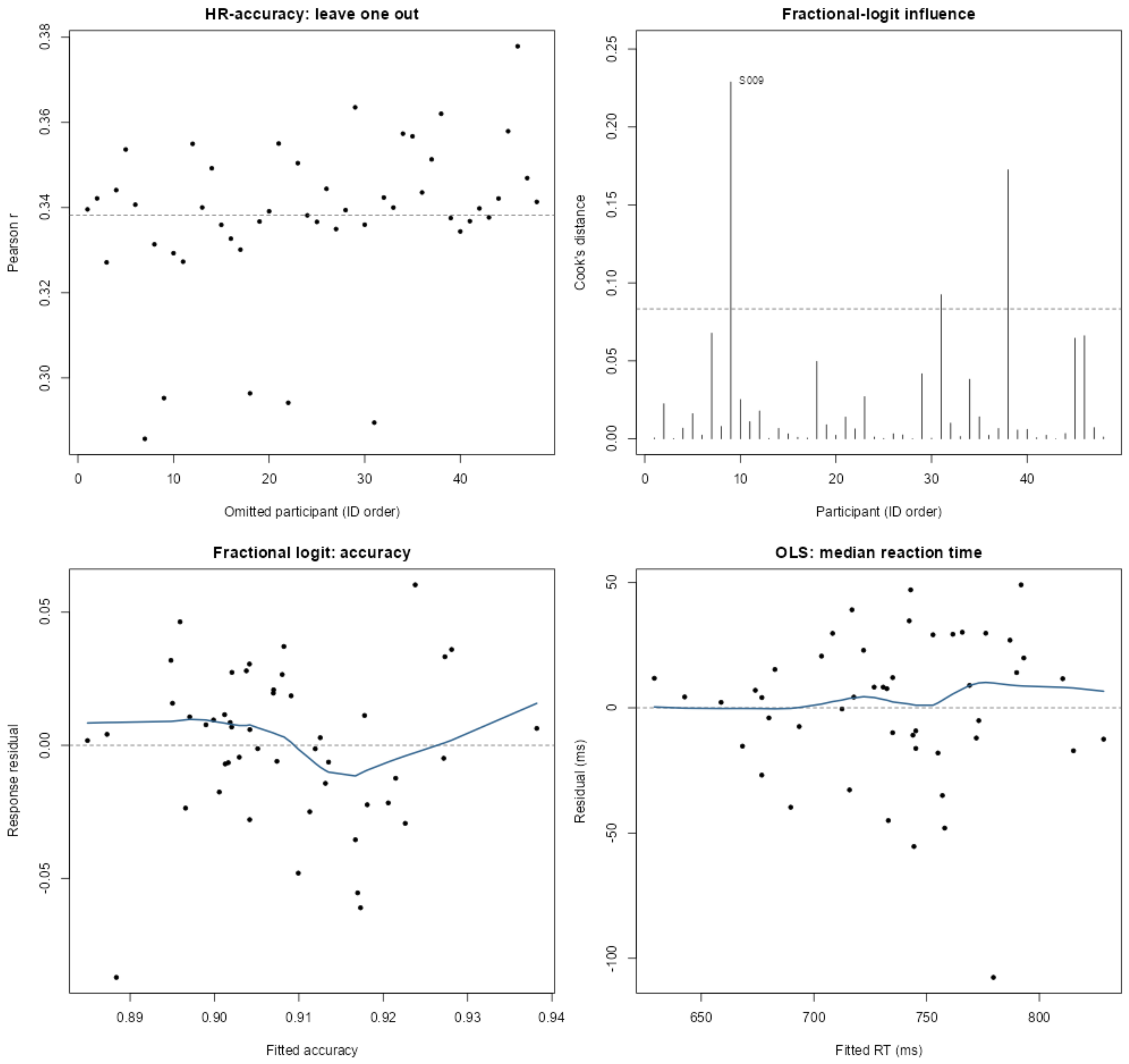

06 | Table S4 negative-binomial offset models

```
Table S4: negative-binomial models with log(DT_Stimuli) offset
 change      outcome  n       IRR   HC3_low  HC3_high    theta Pearson_dispersion df
```

```
      HR   DT_Omitted 48 0.8517137 0.7431016 0.9762005 8.019156          0.9773886 45
      HR DT_Incorrect 48 0.8414060 0.6838706 1.0352310 5.236462          1.1623978 45
      SC   DT_Omitted 48 0.9891177 0.9479988 1.0320200 7.237140          0.8992488 45
      SC DT_Incorrect 48 0.9979475 0.9408165 1.0585477 4.957170          1.1904381 45

HR per 10 bpm; SC per 1 uS. HC3 t intervals; each model includes corresponding baseline.
These are sensitivity estimates; no additional discovery family is defined.

All negative-binomial coefficients; p values unadjusted
          model             term      estimate      SE_HC3 df      CI_low      CI_high            p
   HR DT_Omitted     (Intercept) -2.414058e+00 0.426861931 45 -3.27380177 -1.554313651 1.011262e-06
   HR DT_Omitted     Delta_HR_10 -1.605049e-01 0.067731175 45 -0.29692244 -0.024087264 2.214927e-02
   HR DT_Omitted HR_Baseline_bpm -2.310028e-03 0.005055718 45 -0.01249277  0.007872711 6.499310e-01
 HR DT_Incorrect     (Intercept) -2.881949e+00 0.542408699 45 -3.97441606 -1.789481667 3.220485e-06
 HR DT_Incorrect     Delta_HR_10 -1.726810e-01 0.102926981 45 -0.37998655  0.034624609 1.003384e-01
 HR DT_Incorrect HR_Baseline_bpm  1.481030e-06 0.006024225 45 -0.01213193  0.012134893 9.998049e-01
   SC DT_Omitted     (Intercept) -2.808903e+00 0.128328614 45 -3.06736985 -2.550435662 1.264764e-25
   SC DT_Omitted    SC_Change_uS -1.094199e-02 0.021081354 45 -0.05340202  0.031518036 6.062788e-01
   SC DT_Omitted  SC_Baseline_uS -1.096823e-02 0.023424549 45 -0.05814769  0.036211233 6.418756e-01
 SC DT_Incorrect     (Intercept) -3.105260e+00 0.243157175 45 -3.59500414 -2.615516755 1.445587e-16
 SC DT_Incorrect    SC_Change_uS -2.054651e-03 0.029269869 45 -0.06100719  0.056897891 9.443480e-01
 SC DT_Incorrect  SC_Baseline_uS -2.678869e-02 0.034274593 45 -0.09582126  0.042243885 4.385484e-01
```

07 | Table S5 Williams tests and BCa bootstrap

```
Table S5: dependent overlapping correlations, HR minus SC
      outcome        r_HR        r_SC  difference     BCa_low  BCa_high  Williams_t df         p     B    q_BH_5
 Accuracy_pct  0.33817651  0.07825163  0.25992488 -0.06802843 0.6126756  1.38873716 45 0.1717483 19999 0.5990651
   DT_Omitted -0.29079273 -0.09195306 -0.19883967 -0.59398696 0.1858294 -1.04746389 45 0.3004793 19999 0.5990651
 DT_Incorrect -0.20924178 -0.04788352 -0.16135827 -0.46336727 0.1540675 -0.83354089 45 0.4089424 19999 0.5990651
   DT_Correct  0.18064218  0.04182244  0.13881974 -0.14924635 0.4383453  0.71344868 45 0.4792521 19999 0.5990651
     DT_RT_ms  0.09206541  0.07812559  0.01393982 -0.33570592 0.3747517  0.07097543 45 0.9437318 19999 0.9437318

Williams t tests; BH family of five. BCa: boot::boot and boot::boot.ci, 19999 paired resamples.
Seed 4596147 reset for each outcome. All three variables remain paired in every resample.
```

08 | Table S6 exposure correlations and software versions

```
Table S6: HR-change correlations with aggregate exposure summaries; Fisher-z CI
               summary  n          r     CI_low   CI_high
            DT_Stimuli 48 0.08322372 -0.2057769 0.3588723
              DT_Total 48 0.03833091 -0.2485103 0.3189914
 Response_events_proxy 48 0.10162841 -0.1879331 0.3749364
 Task_phase_duration_s 48 0.09950192 -0.1900046 0.3730887

Correct + incorrect is a response-count proxy. Phase duration is not a verified stimulus-presentation timeline.

Reproduction environment
R version 4.4.1 (2024-06-14 ucrt)
Platform: x86_64-w64-mingw32/x64
Running under: Windows 11 x64 (build 26200)

Matrix products: default


locale:
[1] C
system code page: 65001

time zone: Europe/Warsaw
tzcode source: internal

attached base packages:
[1] stats     graphics  grDevices utils     datasets  methods   base

loaded via a namespace (and not attached):
 [1] vctrs_0.6.5       cli_3.6.3         rlang_1.1.4       generics_0.1.3    textshaping_0.4.0 jsonlite_1.8.8    zoo_1.8-12
 [8] glue_1.7.0        colorspace_2.1-1  ragg_1.3.2        fansi_1.0.6       scales_1.3.0      grid_4.4.1        tibble_3.2.1
[15] evaluate_0.24.0   munsell_0.5.1     MASS_7.3-60.2     fastmap_1.2.0     base64enc_0.1-3   lifecycle_1.0.4   compiler_4.4.1
[22] dplyr_1.1.4       sandwich_3.1-0    pkgconfig_2.0.3   Rcpp_1.0.13       systemfonts_1.1.0 lattice_0.22-6    RProtoBuf_0.4.22
[29] R6_2.5.1          tidyselect_1.2.1  utf8_1.2.4        pillar_1.9.0      magrittr_2.0.3    jmvcore_2.6.3     tools_4.4.1
[36] gtable_0.3.5      boot_1.3-30       Rj_2.7.18         ggplot2_3.5.1
```

09 | Excel EDA_60s reconciliation

```
Excel EDA_60s versus participant summaries: 96 phase comparisons
 Subject_ID    phase windows summed_SCR participant_SCR summed_exposure_s participant_exposure_s reconstructed_per_min participant_per_min
       S001 Baseline       6         22              22          291.4407               291.4407             4.5292231           4.5292231
       S001     Task       9         89              89          486.5000               486.5000            10.9763618          10.9763618
       S002 Baseline       6         32              32          303.3750               303.3750             6.3288010           6.3288010
       S002     Task       9        154             154          491.5000               491.5000            18.7995931          18.7995931
       S003 Baseline       6         67              67          295.4765               295.4765            13.6051414          13.6051414
       S003     Task       9        138             138          486.8750               486.8750            17.0064185          17.0064185
       S004 Baseline       6         30              30          300.2500               300.2500             5.9950042           5.9950042
       S004     Task       9        137             137          492.5000               492.5000            16.6903553          16.6903553
       S005 Baseline       6         53              53          315.6250               315.6250            10.0752475          10.0752475
       S005     Task       9        105             105          489.0000               489.0000            12.8834356          12.8834356
       S006 Baseline       6         23              23          293.1435               293.1435             4.7075916           4.7075916
       S006     Task       9        103             103          487.5000               487.5000            12.6769231          12.6769231
       S007 Baseline       6         53              53          294.4824               294.4824            10.7986098          10.7986098
       S007     Task       9        133             133          490.2500               490.2500            16.2774095          16.2774095
       S008 Baseline       6          9               9          293.2266               293.2266             1.8415793           1.8415793
       S008     Task       9        129             129          490.0000               490.0000            15.7959184          15.7959184
       S009 Baseline       6          8               8          291.9021               291.9021             1.6443871           1.6443871
       S009     Task       9         46              46          487.8750               487.8750             5.6571868           5.6571868
       S010 Baseline       6         10              10          292.1826               292.1826             2.0535104           2.0535104
       S010     Task       9         48              48          493.2500               493.2500             5.8388241           5.8388241
       S011 Baseline       6         28              28          301.4166               301.4166             5.5736817           5.5736817
       S011     Task       9         86              86          500.3843               500.3843            10.3120731          10.3120731
       S012 Baseline       6         25              25          292.1491               292.1491             5.1343650           5.1343650
       S012     Task       9         77              77          491.1250               491.1250             9.4069738           9.4069738
       S013 Baseline       6         36              36          301.7685               301.7685             7.1578046           7.1578046
       S013     Task       9        120             120          499.2792               499.2792            14.4207895          14.4207895
```

S014	Baseline	6	48	48	291.3782	291.3782	9.8840611	9.8840611
S014	Task	9	180	180	475.0000	475.0000	22.7368421	22.7368421
S015	Baseline	6	28	28	291.3786	291.3786	5.7656954	5.7656954
S015	Task	9	92	92	489.0000	489.0000	11.2883436	11.2883436
S016	Baseline	6	11	11	304.8750	304.8750	2.1648216	2.1648216
S016	Task	9	60	60	487.6250	487.6250	7.3827224	7.3827224
S017	Baseline	6	20	20	293.4441	293.4441	4.0893648	4.0893648
S017	Task	9	99	99	477.7500	477.7500	12.4332810	12.4332810
S018	Baseline	6	24	24	291.9207	291.9207	4.9328462	4.9328462
S018	Task	9	96	96	491.1250	491.1250	11.7281751	11.7281751
S019	Baseline	6	13	13	293.7474	293.7474	2.6553427	2.6553427
S019	Task	9	100	100	490.5000	490.5000	12.2324159	12.2324159
S020	Baseline	6	19	19	291.1600	291.1600	3.9153724	3.9153724
S020	Task	9	82	82	477.7500	477.7500	10.2982732	10.2982732
S021	Baseline	6	51	51	290.4563	290.4563	10.5351486	10.5351486
S021	Task	9	107	107	494.1250	494.1250	12.9926638	12.9926638
S022	Baseline	6	25	25	295.6250	295.6250	5.0739958	5.0739958
S022	Task	9	105	105	490.7500	490.7500	12.8374936	12.8374936
S023	Baseline	5	23	23	286.2500	286.2500	4.8209607	4.8209607
S023	Task	9	104	104	490.5000	490.5000	12.7217125	12.7217125
S024	Baseline	6	60	60	292.2466	292.2466	12.3183624	12.3183624
S024	Task	9	127	127	506.7500	506.7500	15.0370005	15.0370005
S025	Baseline	6	43	43	292.1721	292.1721	8.8304105	8.8304105
S025	Task	9	86	86	477.1250	477.1250	10.8147760	10.8147760
S026	Baseline	6	23	23	291.7214	291.7214	4.7305408	4.7305408
S026	Task	9	41	41	489.1250	489.1250	5.0293892	5.0293892
S027	Baseline	6	37	37	299.6250	299.6250	7.4092616	7.4092616
S027	Task	9	97	97	489.8750	489.8750	11.8805818	11.8805818
S028	Baseline	6	18	18	293.1634	293.1634	3.6839520	3.6839520
S028	Task	9	62	62	493.1250	493.1250	7.5437262	7.5437262
S029	Baseline	6	12	12	295.6413	295.6413	2.4353840	2.4353840
S029	Task	9	97	97	490.6250	490.6250	11.8624204	11.8624204
S030	Baseline	6	43	43	293.6547	293.6547	8.7858277	8.7858277
S030	Task	9	81	81	487.7500	487.7500	9.9641210	9.9641210
S031	Baseline	6	16	16	291.9580	291.9580	3.2881446	3.2881446
S031	Task	9	39	39	490.3750	490.3750	4.7718583	4.7718583
S032	Baseline	6	28	28	296.0000	296.0000	5.6756757	5.6756757
S032	Task	9	120	120	489.3750	489.3750	14.7126437	14.7126437
S033	Baseline	6	44	44	291.2386	291.2386	9.0647323	9.0647323
S033	Task	9	131	131	487.2500	487.2500	16.1313494	16.1313494
S034	Baseline	6	18	18	290.4928	290.4928	3.7178204	3.7178204
S034	Task	9	150	150	485.5000	485.5000	18.5375901	18.5375901
S035	Baseline	6	4	4	313.2500	313.2500	0.7661612	0.7661612
S035	Task	9	119	119	489.0000	489.0000	14.6012270	14.6012270
S036	Baseline	6	15	15	298.3750	298.3750	3.0163385	3.0163385
S036	Task	9	97	97	491.3750	491.3750	11.8443144	11.8443144
S037	Baseline	6	13	13	292.6824	292.6824	2.6650047	2.6650047
S037	Task	9	85	85	488.2500	488.2500	10.4454685	10.4454685
S038	Baseline	6	12	12	292.7240	292.7240	2.4596547	2.4596547
S038	Task	9	107	107	489.2500	489.2500	13.1221257	13.1221257
S039	Baseline	6	8	8	293.6453	293.6453	1.6346252	1.6346252
S039	Task	9	24	24	479.7500	479.7500	3.0015633	3.0015633
S040	Baseline	6	15	15	299.8750	299.8750	3.0012505	3.0012505
S040	Task	9	97	97	498.6250	498.6250	11.6720983	11.6720983
S041	Baseline	6	15	15	291.4919	291.4919	3.0875642	3.0875642
S041	Task	9	108	108	488.8750	488.8750	13.2549220	13.2549220
S042	Baseline	6	19	19	303.6250	303.6250	3.7546315	3.7546315
S042	Task	7	90	90	392.7500	392.7500	13.7492043	13.7492043
S043	Baseline	6	36	36	292.4502	292.4502	7.3858739	7.3858739
S043	Task	9	111	111	489.3750	489.3750	13.6091954	13.6091954
S044	Baseline	6	6	6	295.9632	295.9632	1.2163675	1.2163675
S044	Task	9	100	100	488.7500	488.7500	12.2762148	12.2762148
S045	Baseline	6	25	25	295.3750	295.3750	5.0782903	5.0782903
S045	Task	9	87	87	489.6250	489.6250	10.6612203	10.6612203
S046	Baseline	6	1	1	292.4456	292.4456	0.2051664	0.2051664
S046	Task	9	10	10	489.1250	489.1250	1.2266803	1.2266803
S047	Baseline	6	5	5	293.6848	293.6848	1.0215035	1.0215035
S047	Task	9	9	9	488.6250	488.6250	1.1051420	1.1051420
S048	Baseline	6	11	11	301.0000	301.0000	2.1926910	2.1926910
S048	Task	9	57	57	487.0000	487.0000	7.0225873	7.0225873

last_cumulative	sum_interval_duration_s	phase_duration_s	count_difference	exposure_difference_s	rate_difference
22	300.6907	300.6907	0	0	0.000000e+00
89	496.5008	496.5008	0	0	0.000000e+00
32	313.3739	313.3739	0	0	0.000000e+00
154	501.5767	501.5767	0	0	0.000000e+00
67	302.7265	302.7265	0	0	0.000000e+00
138	496.9020	496.9020	0	0	0.000000e+00
30	310.1540	310.1540	0	0	0.000000e+00
137	502.5295	502.5295	0	0	0.000000e+00
53	325.5230	325.5230	0	0	0.000000e+00
105	499.0612	499.0612	0	0	0.000000e+00
23	301.5185	301.5185	0	0	0.000000e+00
103	497.4837	497.4837	0	0	0.000000e+00
53	302.2324	302.2324	0	0	0.000000e+00
133	500.2620	500.2620	0	0	0.000000e+00
9	301.6016	301.6016	0	0	0.000000e+00
129	499.9903	499.9903	0	0	0.000000e+00
8	300.9021	300.9021	0	0	0.000000e+00
46	497.9436	497.9436	0	0	0.000000e+00
10	301.0576	301.0576	0	0	0.000000e+00
48	503.2924	503.2924	0	0	0.000000e+00
28	301.4166	301.4166	0	0	8.881784e-16
86	500.3843	500.3843	0	0	0.000000e+00
25	301.0241	301.0241	0	0	0.000000e+00
77	501.1144	501.1144	0	0	0.000000e+00
36	301.7685	301.7685	0	0	8.881784e-16
120	499.2792	499.2792	0	0	0.000000e+00
48	300.6282	300.6282	0	0	0.000000e+00
180	497.5178	497.5178	0	0	0.000000e+00
28	300.6286	300.6286	0	0	0.000000e+00
92	498.9917	498.9917	0	0	0.000000e+00
11	314.8372	314.8372	0	0	0.000000e+00
60	497.6830	497.6830	0	0	0.000000e+00
20	301.6941	301.6941	0	0	0.000000e+00
99	497.8579	497.8579	0	0	0.000000e+00

24	300.9207	300.9207	0	0	0.000000e+00
96	501.1351	501.1351	0	0	0.000000e+00
13	301.8724	301.8724	0	0	0.000000e+00
100	500.5675	500.5675	0	0	0.000000e+00
19	300.5350	300.5350	0	0	0.000000e+00
82	498.3096	498.3096	0	0	0.000000e+00
51	300.2063	300.2063	0	0	0.000000e+00
107	504.1393	504.1393	0	0	0.000000e+00
25	305.5411	305.5411	0	0	0.000000e+00
105	500.6682	500.6682	0	0	0.000000e+00
23	296.1694	296.1694	0	0	0.000000e+00
104	500.5301	500.5301	0	0	0.000000e+00
60	301.1216	301.1216	0	0	0.000000e+00
127	516.7521	516.7521	0	0	0.000000e+00
43	301.0471	301.0471	0	0	0.000000e+00
86	497.1296	497.1296	0	0	0.000000e+00
23	300.8464	300.8464	0	0	0.000000e+00
41	499.1557	499.1557	0	0	0.000000e+00
37	309.5340	309.5340	0	0	0.000000e+00
97	499.8846	499.8846	0	0	0.000000e+00
18	301.5384	301.5384	0	0	0.000000e+00
62	503.1411	503.1411	0	0	0.000000e+00
12	302.7663	302.7663	0	0	0.000000e+00
97	500.6297	500.6297	0	0	0.000000e+00
43	301.7797	301.7797	0	0	0.000000e+00
81	497.7503	497.7503	0	0	0.000000e+00
16	300.9580	300.9580	0	0	0.000000e+00
39	500.3100	500.3100	0	0	0.000000e+00
28	305.8991	305.8991	0	0	0.000000e+00
120	499.4454	499.4454	0	0	0.000000e+00
44	300.6136	300.6136	0	0	0.000000e+00
131	497.2557	497.2557	0	0	0.000000e+00
18	300.2428	300.2428	0	0	0.000000e+00
150	495.5906	495.5906	0	0	0.000000e+00
4	323.2218	323.2218	0	0	0.000000e+00
119	499.0641	499.0641	0	0	0.000000e+00
15	308.3453	308.3453	0	0	0.000000e+00
97	501.4595	501.4595	0	0	0.000000e+00
13	301.3074	301.3074	0	0	0.000000e+00
85	498.3559	498.3559	0	0	0.000000e+00
12	301.3490	301.3490	0	0	0.000000e+00
107	499.1972	499.1972	0	0	0.000000e+00
8	301.7703	301.7703	0	0	0.000000e+00
24	499.7312	499.7312	0	0	0.000000e+00
15	309.7925	309.7925	0	0	0.000000e+00
97	508.5125	508.5125	0	0	0.000000e+00
15	300.7419	300.7419	0	0	0.000000e+00
108	498.9342	498.9342	0	0	0.000000e+00
19	313.6205	313.6205	0	0	0.000000e+00
90	402.7654	402.7654	0	0	0.000000e+00
36	301.2002	301.2002	0	0	0.000000e+00
111	499.3177	499.3177	0	0	0.000000e+00
6	302.9632	302.9632	0	0	0.000000e+00
100	498.7310	498.7310	0	0	0.000000e+00
25	305.2793	305.2793	0	0	0.000000e+00
87	499.6105	499.6105	0	0	0.000000e+00
1	301.1956	301.1956	0	0	0.000000e+00
10	499.2259	499.2259	0	0	0.000000e+00
5	301.8098	301.8098	0	0	0.000000e+00
9	498.5608	498.5608	0	0	0.000000e+00
11	310.9168	310.9168	0	0	0.000000e+00
57	497.0894	497.0894	0	0	0.000000e+00

Reconciliation flags (no automatic replacement or exclusion)

check	phase_count
SCR count differs	0
Cumulative count differs	0
Exposure differs > 0.13 s	0
Rate differs > 0.01/min	0

Analysis unit remains the participant. Window rows are used only for reconciliation.
No raw filtering, cvxEDA decomposition or peak detection was rerun.
Phase-level SCL and amplitude remain the recorded Participant_results values.

Native | Descriptive statistics

Descriptives

	N	Missing	Mean	95% Confidence Interval Lower	95% Confidence Interval Upper	Median	SD	IQR	Minimum	Maximum
**HR_Baseline_bpm**	48	0	75.7775	72.6014	78.9535	76.6465	10.9379	12.2861	48.9870	100.092
**HR_Task_bpm**	48	0	95.7666	92.0315	99.5017	94.8288	12.8632	20.5783	74.2590	121.923
**HR_Change_bpm**	48	0	19.9891	17.6294	22.3489	18.4619	8.1267	9.1366	8.0940	48.093
**SC_Baseline_uS**	48	0	3.9881	3.1757	4.8005	3.5210	2.7978	2.9100	0.6205	12.322
**SC_Task_uS**	48	0	8.6421	7.4273	9.8568	7.4060	4.1835	6.1039	2.5109	17.326
**SC_Change_uS**	48	0	4.6540	3.9161	5.3918	3.9171	2.5411	3.9082	0.6020	10.637
**DT_Correct**	48	0	522.0417	508.8984	535.1849	523.0000	45.2637	64.7500	417	596
**DT_Incorrect**	48	0	22.6875	18.6881	26.6869	21.0000	13.7735	13.5000	3	89
**DT_Omitted**	48	0	30.5208	27.1928	33.8488	31.5000	11.4613	14.2500	6	54
**DT_Total**	48	0	575.2500	560.3895	590.1105	567.5000	51.1778	78.2500	483	689
**DT_Stimuli**	48	0	559.0833	546.5085	571.6582	554.0000	43.3063	65.0000	474	650
**DT_Accuracy**	48	0	0.9081	0.8992	0.9170	0.9090	0.0308	0.0337	0.8012	0.984
**DT_Omission_proportion**	48	0	0.0534	0.0475	0.0594	0.0542	0.0206	0.0278	0.0106	0.101
**DT_RT_median_s**	48	0	0.7346	0.7189	0.7504	0.7355	0.0543	0.0915	0.6410	0.841

Uwaga. The CI of the mean assumes sample means follow a t-distribution with N - 1 degrees of freedom

Native | EDA summaries

Descriptives

	N	Missing	Mean	Median	SD	IQR	Minimum	Maximum
**Baseline_SCL_uS**	48	0	3.4808	3.0986	2.5513	2.2941	0.5898	11.355
**Task_SCL_uS**	48	0	7.8546	6.8153	4.2326	6.2476	1.2772	16.975
**Delta_SCL_uS**	48	0	4.3738	3.6730	2.5207	3.7102	0.5970	10.139
**Baseline_SCR_model_per_min**	48	0	5.0147	4.6184	3.2003	3.9296	0.2052	13.605
**Task_SCR_model_per_min**	48	0	11.6098	12.0565	4.4242	3.7024	1.1051	22.737
**Delta_SCR_model_per_min**	48	0	6.5951	6.4150	3.8196	5.2933	0.0836	14.820
**Baseline_SCR_amplitude_zero_coded_uS**	48	0	0.1795	0.1722	0.0906	0.0974	0.0559	0.470
**Task_SCR_amplitude_zero_coded_uS**	48	0	0.2350	0.2255	0.0880	0.1011	0.0622	0.500
**Delta_SCR_amplitude_zero_coded_uS**	48	0	0.0555	0.0528	0.1001	0.0906	-0.2162	0.415
**Baseline_EDA_model_exposure_s**	48	0	295.4411	293.3353	5.6735	4.6451	286.2500	315.625
**Task_EDA_model_exposure_s**	48	0	487.2378	489.1875	15.0051	3.2500	392.7500	506.750

Native | HR_Change_bpm Pearson + Spearman (unadjusted p)

Correlation Matrix

		HR_Change_bpm	DT_Accuracy	DT_Omitted	DT_Incorrect	DT_Correct	DT_RT_median_s
**HR_Change_bpm**	**Pearson's r**	—					
	**df**	—					
	**p-value**	—					
	**95% CI Upper**	—					
	**95% CI Lower**	—					
	**Spearman's rho**	—					
	**df**	—					
	**p-value**	—					
	**N**	—					
**DT_Accuracy**	**Pearson's r**	0.338	—				
	**df**	46	—				
	**p-value**	0.019	—				
	**95% CI Upper**	0.568	—				
	**95% CI Lower**	0.060	—				
	**Spearman's rho**	0.192	—				
	**df**	46	—				
	**p-value**	0.190	—				
	**N**	48	—				
**DT_Omitted**	**Pearson's r**	-0.291	-0.840	—			
	**df**	46	46	—			
	**p-value**	0.045	<.001	—			
	**95% CI Upper**	-0.007	-0.731	—			
	**95% CI Lower**	-0.531	-0.908	—			
	**Spearman's rho**	-0.215	-0.866	—			
	**df**	46	46	—			
	**p-value**	0.142	<.001	—			
	**N**	48	48	—			
**DT_Incorrect**	**Pearson's r**	-0.209	-0.707	0.273	—		
	**df**	46	46	46	—		
	**p-value**	0.153	<.001	0.060	—		
	**95% CI Upper**	0.080	-0.530	0.517	—		
	**95% CI Lower**	-0.466	-0.826	-0.012	—		
	**Spearman's rho**	-0.081	-0.574	0.188	—		
	**df**	46	46	46	—		
	**p-value**	0.583	<.001	0.201	—		
	**N**	48	48	48	—		
**DT_Correct**	**Pearson's r**	0.181	0.174	-0.301	0.381	—	
	**df**	46	46	46	46	—	
	**p-value**	0.219	0.237	0.037	0.007	—	
	**95% CI Upper**	0.442	0.436	-0.019	0.600	—	
	**95% CI Lower**	-0.109	-0.116	-0.539	0.109	—	
	**Spearman's rho**	0.131	0.195	-0.289	0.413	—	
	**df**	46	46	46	46	—	
	**p-value**	0.373	0.184	0.047	0.004	—	
	**N**	48	48	48	48	—	

Correlation Matrix

**DT_RT_median_s**	**Pearson's r**	**0.092**	**0.323**	**-0.225**	**-0.563**	**-0.698**	**—**
	**df**	**46**	**46**	**46**	**46**	**46**	**—**
	**p-value**	**0.534**	**0.025**	**0.124**	**<.001**	**<.001**	**—**
	**95% CI Upper**	**0.367**	**0.556**	**0.063**	**-0.332**	**-0.516**	**—**
	**95% CI Lower**	**-0.197**	**0.043**	**-0.479**	**-0.731**	**-0.819**	**—**
	**Spearman's rho**	**0.080**	**0.255**	**-0.196**	**-0.580**	**-0.688**	**—**
	**df**	**46**	**46**	**46**	**46**	**46**	**—**
	**p-value**	**0.587**	**0.080**	**0.182**	**<.001**	**<.001**	**—**
	**N**	**48**	**48**	**48**	**48**	**48**	**—**

Native | SC_Change_uS Pearson + Spearman (unadjusted p)

Correlation Matrix

		SC_Change_uS	DT_Accuracy	DT_Omitted	DT_Incorrect	DT_Correct	DT_RT_median_s
SC_Change_uS	Pearson's r	—					
	df	—					
	p-value	—					
	95% CI Upper	—					
	95% CI Lower	—					
	Spearman's rho	—					
	df	—					
	p-value	—					
	N	—					
DT_Accuracy	Pearson's r	0.078	—				
	df	46	—				
	p-value	0.597	—				
	95% CI Upper	0.355	—				
	95% CI Lower	-0.211	—				
	Spearman's rho	0.032	—				
	df	46	—				
	p-value	0.827	—				
	N	48	—				
DT_Omitted	Pearson's r	-0.092	-0.840	—			
	df	46	46	—			
	p-value	0.534	<.001	—			
	95% CI Upper	0.197	-0.731	—			
	95% CI Lower	-0.367	-0.908	—			
	Spearman's rho	-0.065	-0.866	—			
	df	46	46	—			
	p-value	0.661	<.001	—			
	N	48	48	—			
DT_Incorrect	Pearson's r	-0.048	-0.707	0.273	—		
	df	46	46	46	—		
	p-value	0.747	<.001	0.060	—		
	95% CI Upper	0.240	-0.530	0.517	—		
	95% CI Lower	-0.328	-0.826	-0.012	—		
	Spearman's rho	0.071	-0.574	0.188	—		
	df	46	46	46	—		
	p-value	0.632	<.001	0.201	—		
	N	48	48	48	—		
DT_Correct	Pearson's r	0.042	0.174	-0.301	0.381	—	
	df	46	46	46	46	—	
	p-value	0.778	0.237	0.037	0.007	—	
	95% CI Upper	0.322	0.436	-0.019	0.600	—	
	95% CI Lower	-0.245	-0.116	-0.539	0.109	—	
	Spearman's rho	-0.000	0.195	-0.289	0.413	—	
	df	46	46	46	46	—	
	p-value	0.999	0.184	0.047	0.004	—	
	N	48	48	48	48	—	

Correlation Matrix

**DT_RT_median_s**	**Pearson's r**	**0.078**	**0.323**	**-0.225**	**-0.563**	**-0.698**	**—**
	**df**	**46**	**46**	**46**	**46**	**46**	**—**
	**p-value**	**0.598**	**0.025**	**0.124**	**<.001**	**<.001**	**—**
	**95% CI Upper**	**0.354**	**0.556**	**0.063**	**-0.332**	**-0.516**	**—**
	**95% CI Lower**	**-0.211**	**0.043**	**-0.479**	**-0.731**	**-0.819**	**—**
	**Spearman's rho**	**0.119**	**0.255**	**-0.196**	**-0.580**	**-0.688**	**—**
	**df**	**46**	**46**	**46**	**46**	**46**	**—**
	**p-value**	**0.422**	**0.080**	**0.182**	**<.001**	**<.001**	**—**
	**N**	**48**	**48**	**48**	**48**	**48**	**—**

Native reference | Editable OLS | HR Accuracy_pct + baseline; conventional SE (conventional SE)

Model Fit Measures

Model	R	R^2	Adjusted R^2
1	0.341	0.116	0.0767

Uwaga. Models estimated using sample size of N=48

Model Coefficients - Accuracy_pct

			95% Confidence Interval			
Predictor	Estimate	SE	Lower	Upper	t	p
Intercept	87.3501	3.3301	80.6429	94.0572	26.231	<.001
Delta_HR_10	1.2975	0.5340	0.2221	2.3730	2.430	0.019
HR_Baseline_bpm	0.0114	0.0397	-0.0685	0.0913	0.288	0.774

Data Summary

Cook's Distance

			Range	
Mean	Median	SD	Min	Max
0.0206	0.00702	0.0365	1.02e-6	0.190

Assumption Checks

Collinearity Statistics

	VIF	Tolerance
**Delta_HR_10**	1.01	0.987
**HR_Baseline_bpm**	1.01	0.987

[3]

Native reference | Editable OLS | HR DT_Omitted + baseline; conventional SE (conventional SE)

Model Fit Measures

Model	R	R^2	Adjusted R^2
1	0.291	0.0848	0.0441

Uwaga. Models estimated using sample size of N=48

Model Coefficients - DT_Omitted

			95% Confidence Interval			
Predictor	Estimate	SE	Lower	Upper	t	p
Intercept	40.0331	12.625	14.605	65.4609	3.171	0.003
Delta_HR_10	-4.1266	2.024	-8.204	-0.0493	-2.038	0.047
HR_Baseline_bpm	-0.0167	0.150	-0.320	0.2863	-0.111	0.912

Data Summary

Cook's Distance

			Range	
Mean	Median	SD	Min	Max
0.0189	0.00695	0.0229	8.11e-5	0.0796

Assumption Checks

Collinearity Statistics

	VIF	Tolerance
**Delta_HR_10**	1.01	0.987
**HR_Baseline_bpm**	1.01	0.987

[3]

Native reference | Editable OLS | HR DT_Incorrect + baseline; conventional SE (conventional SE)

Model Fit Measures

Model	R	R^2	Adjusted R^2
1	0.213	0.0452	0.00276

Uwaga. Models estimated using sample size of N=48

Model Coefficients - DT_Incorrect

			95% Confidence Interval			
Predictor	Estimate	SE	Lower	Upper	t	p
Intercept	26.0136	15.497	-5.198	57.226	1.679	0.100
Delta_HR_10	-3.4733	2.485	-8.478	1.532	-1.398	0.169
HR_Baseline_bpm	0.0477	0.185	-0.324	0.420	0.259	0.797

Data Summary

Cook's Distance

			Range	
Mean	Median	SD	Min	Max
0.0199	0.00572	0.0566	1.02e-7	0.390

Assumption Checks

Collinearity Statistics

	VIF	Tolerance
**Delta_HR_10**	1.01	0.987
**HR_Baseline_bpm**	1.01	0.987

[3]

Native reference | Editable OLS | HR DT_Correct + baseline; conventional SE (conventional SE)

Model Fit Measures

Model	R	R^2	Adjusted R^2
1	0.269	0.0724	0.0311

Uwaga. Models estimated using sample size of N=48

Model Coefficients - DT_Correct

			95% Confidence Interval			
Predictor	Estimate	SE	Lower	Upper	t	p
Intercept	436.470	50.197	335.368	537.57	8.70	<.001
Delta_HR_10	11.332	8.049	-4.879	27.54	1.41	0.166
HR_Baseline_bpm	0.830	0.598	-0.374	2.03	1.39	0.172

Data Summary

Cook's Distance

			Range	
Mean	Median	SD	Min	Max
0.0193	0.00882	0.0242	2.27e-4	0.109

Assumption Checks

Collinearity Statistics

	VIF	Tolerance
**Delta_HR_10**	1.01	0.987
**HR_Baseline_bpm**	1.01	0.987

[3]

Native reference | Editable OLS | HR DT_RT_ms + baseline; conventional SE (conventional SE)

Model Fit Measures

Model	R	R^2	Adjusted R^2
1	0.244	0.0594	0.0176

Uwaga. Models estimated using sample size of N=48

Model Coefficients - DT_RT_ms

			95% Confidence Interval			
Predictor	Estimate	SE	Lower	Upper	t	p
Intercept	811.28	60.658	689.11	933.450	13.375	<.001
Delta_HR_10	4.43	9.726	-15.16	24.017	0.455	0.651
HR_Baseline_bpm	-1.13	0.723	-2.58	0.327	-1.561	0.125

Data Summary

Cook's Distance

			Range	
Mean	Median	SD	Min	Max
0.0220	0.0138	0.0237	1.04e-7	0.113

Assumption Checks

Collinearity Statistics

	VIF	Tolerance
**Delta_HR_10**	1.01	0.987
**HR_Baseline_bpm**	1.01	0.987

[3]

Native reference | Editable OLS | SC Accuracy_pct + baseline; conventional SE (conventional SE)

Model Fit Measures

Model	R	R^2	Adjusted R^2
1	0.158	0.0249	-0.0185

Uwaga. Models estimated using sample size of N=48

Model Coefficients - Accuracy_pct

			95% Confidence Interval			
Predictor	Estimate	SE	Lower	Upper	t	p
Intercept	89.9327	1.053	87.811	92.054	85.382	<.001
SC_Change_uS	0.0562	0.183	-0.312	0.425	0.307	0.760
SC_Baseline_uS	0.1545	0.166	-0.180	0.489	0.930	0.357

Data Summary

Cook's Distance

			Range	
Mean	Median	SD	Min	Max
0.0192	0.00785	0.0378	1.63e-5	0.225

Assumption Checks

Collinearity Statistics

	VIF	Tolerance
**SC_Change_uS**	1.05	0.949
**SC_Baseline_uS**	1.05	0.949

[3]

Native reference | Editable OLS | SC DT_Omitted + baseline; conventional SE (conventional SE)

Model Fit Measures

Model	R	R^2	Adjusted R^2
1	0.127	0.0162	-0.0275

Uwaga. Models estimated using sample size of N=48

Model Coefficients - DT_Omitted

			95% Confidence Interval			
Predictor	Estimate	SE	Lower	Upper	t	p
Intercept	33.501	3.942	25.56	41.441	8.499	<.001
SC_Change_uS	-0.322	0.685	-1.70	1.057	-0.471	0.640
SC_Baseline_uS	-0.371	0.622	-1.62	0.881	-0.597	0.553

Data Summary

Cook's Distance

			Range	
Mean	Median	SD	Min	Max
0.0223	0.00883	0.0380	3.58e-7	0.234

Assumption Checks

Collinearity Statistics

	VIF	Tolerance
**SC_Change_uS**	1.05	0.949
**SC_Baseline_uS**	1.05	0.949

[3]

Native reference | Editable OLS | SC DT_Incorrect + baseline; conventional SE (conventional SE)

Model Fit Measures

Model	R	R^2	Adjusted R^2
1	0.147	0.0216	-0.0219

Uwaga. Models estimated using sample size of N=48

Model Coefficients - DT_Incorrect

			95% Confidence Interval			
Predictor	Estimate	SE	Lower	Upper	t	p
Intercept	25.8818	4.724	16.37	35.397	5.479	<.001
SC_Change_uS	-0.0847	0.820	-1.74	1.568	-0.103	0.918
SC_Baseline_uS	-0.7022	0.745	-2.20	0.799	-0.942	0.351

Data Summary

Cook's Distance

			Range	
Mean	Median	SD	Min	Max
0.0229	0.00700	0.0728	2.33e-6	0.450

Assumption Checks

Collinearity Statistics

	VIF	Tolerance
**SC_Change_uS**	1.05	0.949
**SC_Baseline_uS**	1.05	0.949

[3]

Native reference | Editable OLS | SC DT_Correct + baseline; conventional SE (conventional SE)

Model Fit Measures

Model	R	R^2	Adjusted R^2
1	0.0983	0.00966	-0.0344

Uwaga. Models estimated using sample size of N=48

Model Coefficients - DT_Correct

			95% Confidence Interval			
Predictor	Estimate	SE	Lower	Upper	t	p
Intercept	522.75	15.62	491.29	554.21	33.468	<.001
SC_Change_uS	1.11	2.71	-4.35	6.58	0.410	0.684
SC_Baseline_uS	-1.48	2.46	-6.44	3.49	-0.600	0.552

Data Summary

Cook's Distance

			Range	
Mean	Median	SD	Min	Max
0.0244	0.0103	0.0546	7.98e-5	0.369

Assumption Checks

Collinearity Statistics

	VIF	Tolerance
**SC_Change_uS**	1.05	0.949
**SC_Baseline_uS**	1.05	0.949

[3]

Native reference | Editable OLS | SC DT_RT_ms + baseline; conventional SE (conventional SE)

Model Fit Measures

Model	R	R^2	Adjusted R^2
1	0.0838	0.00703	-0.0371

Uwaga. Models estimated using sample size of N=48

Model Coefficients - DT_RT_ms

			95% Confidence Interval			
Predictor	Estimate	SE	Lower	Upper	t	p
Intercept	725.141	18.77	687.34	762.94	38.634	<.001
SC_Change_uS	1.519	3.26	-5.05	8.08	0.466	0.643
SC_Baseline_uS	0.605	2.96	-5.36	6.57	0.204	0.839

Data Summary

Cook's Distance

			Range	
Mean	Median	SD	Min	Max
0.0306	0.0153	0.0590	1.05e-5	0.323

Assumption Checks

Collinearity Statistics

	VIF	Tolerance
**SC_Change_uS**	1.05	0.949
**SC_Baseline_uS**	1.05	0.949

[3]

Bibliografia

[1] The jamovi project (2024). *jamovi*. (Version 2.6) [Computer Software]. Retrieved from https://www.jamovi.org.

[2] R Core Team (2024). *R: A Language and environment for statistical computing*. (Version 4.4) [Computer software]. Retrieved from https://cran.r-project.org. (R packages retrieved from CRAN snapshot 2024-08-07).

[3] Fox, J., & Weisberg, S. (2023). *car: Companion to Applied Regression*. [R package]. Retrieved from https://cran.r-project.org/package=car.
