# Supplementary material for: Autonomic Changes During an Adaptive Cognitive Task
Source: J Clin Med. 2026 Oct 17;15(20):8068. doi: 10.3390/jcm15208068 (PMC13705764; doi:10.3390/jcm15208068)
Supplement: Supplementary file 1 [file jcm-15-08068-s001.zip › Supplementary_File_S3_Tables_and_Figures.pdf]

These tables accompany the manuscript. All main models use 48 participants. The primary family contains ten Pearson tests; complementary models are exploratory. Individual confidence intervals are reported separately from the stated BH families. Bootstrap analyses used R 4.4.1 with seed 4,596,147 reset for each model or comparison and participants ordered by Subject_ID.

Table S1 Baseline adjusted OLS sensitivity analyses

Change	Outcome	Slope [95% HC3 CI]	p	BH q
ΔHR	Accuracy	1.298 [0.163, 2.432]	0.026	0.164
ΔHR	Omitted count	−4.127 [−7.901, −0.352]	0.033	0.164
ΔHR	Incorrect count	−3.473 [−8.847, 1.900]	0.200	0.499
ΔHR	Correct count	11.332 [−2.268, 24.932]	0.100	0.334
ΔHR	Median RT	4.427 [−15.248, 24.101]	0.653	0.808
ΔSC	Accuracy	0.056 [−0.266, 0.379]	0.727	0.808
ΔSC	Omitted count	−0.322 [−1.657, 1.013]	0.629	0.808
ΔSC	Incorrect count	−0.085 [−1.562, 1.393]	0.909	0.909
ΔSC	Correct count	1.113 [−3.860, 6.085]	0.654	0.808
ΔSC	Median RT	1.519 [−6.019, 9.058]	0.687	0.808

Each model contains physiological change and its corresponding baseline value (n = 48; residual df = 45). HR slopes are per 10 bpm and SC slopes per 1 µS. Accuracy is in percentage points, counts in responses, and RT in ms. BH correction covers these ten focal change coefficients. HC3 results are provided in Supplementary File S2.

Table S2 Rank robust slope and leave one out analyses

Change / outcome	ρ	p	BH q	Leave-one-out r	Theil–Sen slope [95% CI]
ΔHR / Accuracy	0.192	0.190	0.826	0.286 to 0.378	0.76 [−0.50, 2.20]
ΔHR / Omitted count	−0.215	0.142	0.826	−0.329 to −0.238	−3.80 [−9.11, 0.91]
ΔHR / Incorrect count	−0.081	0.583	0.826	−0.249 to −0.125	−0.94 [−5.70, 4.35]
ΔHR / Correct count	0.131	0.373	0.826	0.102 to 0.232	9.58 [−9.83, 26.90]
ΔHR / Median RT	0.080	0.587	0.826	0.046 to 0.130	5.79 [−18.88, 33.57]
ΔSC / Accuracy	0.032	0.827	0.919	0.010 to 0.134	0.02 [−0.34, 0.36]
ΔSC / Omitted count	−0.065	0.661	0.826	−0.141 to −0.053	−0.31 [−1.86, 1.45]
ΔSC / Incorrect count	0.071	0.632	0.826	−0.087 to 0.070	0.19 [−1.30, 1.45]
ΔSC / Correct count	0.000	0.999	0.999	−0.020 to 0.088	0.00 [−4.99, 5.36]
ΔSC / Median RT	0.119	0.422	0.826	0.001 to 0.148	2.74 [−3.88, 10.87]

Spearman p values are two-sided; q covers ten rank tests. Pearson leave-one-out ranges omit each participant once (n = 47). Theil–Sen 95% percentile intervals use 1999 paired participant bootstrap resamples and R type 7 quantiles. Slope units match Table S1; these are unadjusted bivariate slopes.

Table S3 Sensitivity of the HR accuracy model

Specification	n	AME (pp/10 bpm)	Logit coefficient [95% HC3 CI]
Main adjusted model	48	1.419	0.170 [0.014, 0.327]
Add sex and KSS	48	1.396	0.168 [0.012, 0.323]
Add sex and sleep duration	48	1.370	0.164 [0.014, 0.315]
Replace DT_Stimuli with DT_Total	48	1.407	0.169 [0.021, 0.317]

The starting fractional-logit model includes HR change, baseline HR, and DT_Stimuli. The AME is an average marginal derivative. The displayed confidence interval is for the logit coefficient, not for the AME.

For HR and accuracy, the AME was 1.371 percentage points per 10 bpm before inclusion of DT_Stimuli and 1.419 afterward. Across all 48 leave-one-out fits, the adjusted AME ranged from 1.054 to 1.603. All 4999 bootstrap replicates converged for each of the 15 complementary models. The HR baseline-by-change interaction was 0.0009 on the logit scale (95% HC3 CI −0.1662 to 0.1679; p = 0.992; BH q = 0.992).

Table S4 Negative binomial models with DT_Stimuli offset

Change	Outcome	IRR [95% HC3 CI]	θ	Pearson dispersion
ΔHR	Omitted count	0.852 [0.743, 0.976]	8.02	0.98
ΔHR	Incorrect count	0.841 [0.684, 1.035]	5.24	1.16
ΔSC	Omitted count	0.989 [0.948, 1.032]	7.24	0.90
ΔSC	Incorrect count	0.998 [0.941, 1.059]	4.96	1.19

Each model contains physiological change and its corresponding baseline, with log(DT_Stimuli) as the offset (n = 48). HR effects are per 10 bpm and SC effects per 1 μS. These estimates describe expected count ratios at fixed exposure. They are sensitivity analyses and are not additional multiplicity-adjusted discoveries. θ is the estimated negative-binomial size parameter.

Table S5 Direct comparison of HR and SC correlations

Outcome	Difference in r [95% BCa CI]	Williams t	p	BH q
Accuracy	0.260 [−0.068, 0.613]	1.389	0.172	0.599
Omitted count	−0.199 [−0.594, 0.186]	−1.047	0.300	0.599
Incorrect count	−0.161 [−0.463, 0.154]	−0.834	0.409	0.599
Correct count	0.139 [−0.149, 0.438]	0.713	0.479	0.599
Median RT	0.014 [−0.336, 0.375]	0.071	0.944	0.944

Difference = HR correlation minus SC correlation for the same outcome. BCa intervals use 19,999 paired participant resamples and the boot::boot.ci implementation. Williams tests have 45 degrees of freedom. BH correction covers the five comparisons. Every interval includes zero.

Table S6 HR associations with aggregate exposure summaries

Summary	Pearson r [95% CI]
DT_Stimuli	0.083 [−0.206, 0.359]
Correct + incorrect + omitted	0.038 [−0.249, 0.319]
Correct + incorrect	0.102 [−0.188, 0.375]
Physiological task-phase duration	0.100 [−0.190, 0.373]

n = 48. These descriptive correlations use Fisher-z intervals. Correct + incorrect is a response-count proxy, not an event-by-event motor record.

Influence and residual diagnostics

Cook's distance, leverage, variance-inflation factors, residual plots, and leave-one-out estimates were examined. In the adjusted HR–accuracy model, S009 had the largest Cook's distance. The leave-one-out results preserve the positive direction but show meaningful variation in magnitude; they are not a reason to remove the observation. The alternative additive parameterizations change + baseline and task + baseline were numerically equivalent.

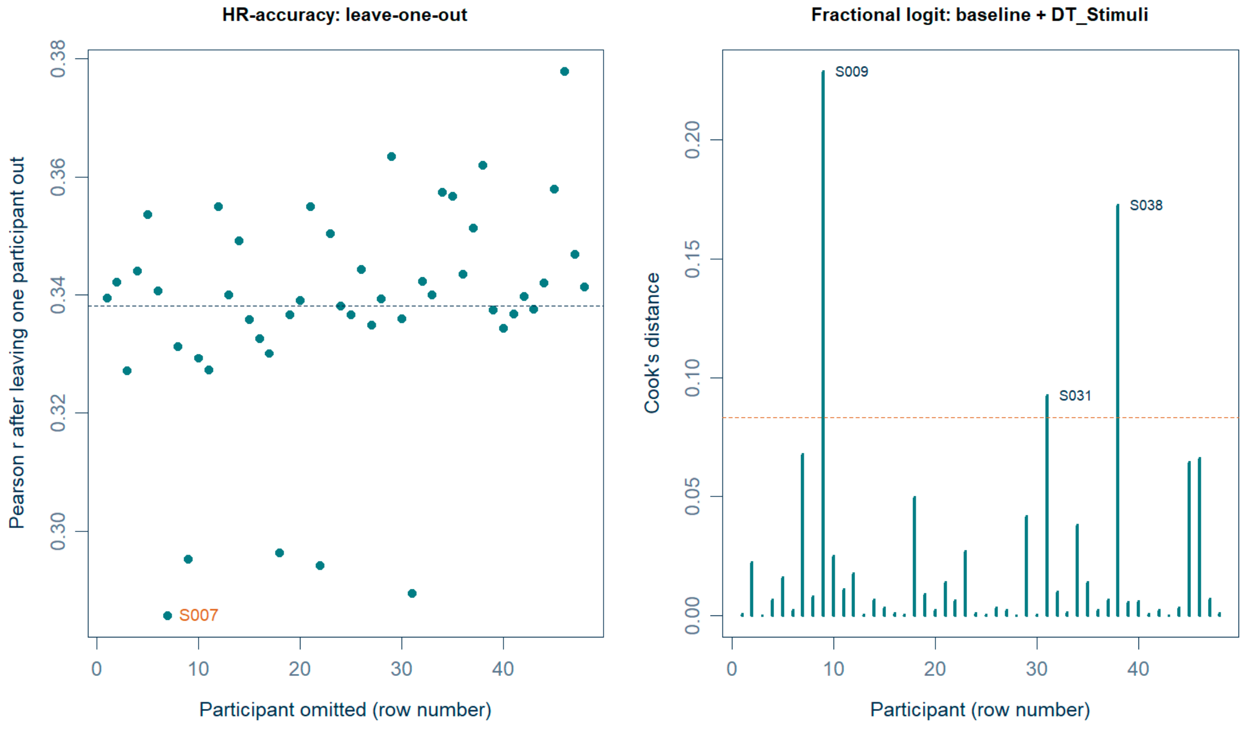

Figure S1. Pearson HR–accuracy leave-one-out estimates (left) and Cook's distance for the accuracy fractional-logit model with baseline HR and DT_Stimuli (right). The horizontal Cook's-distance line is 4/n, used as a screening reference rather than an exclusion rule.

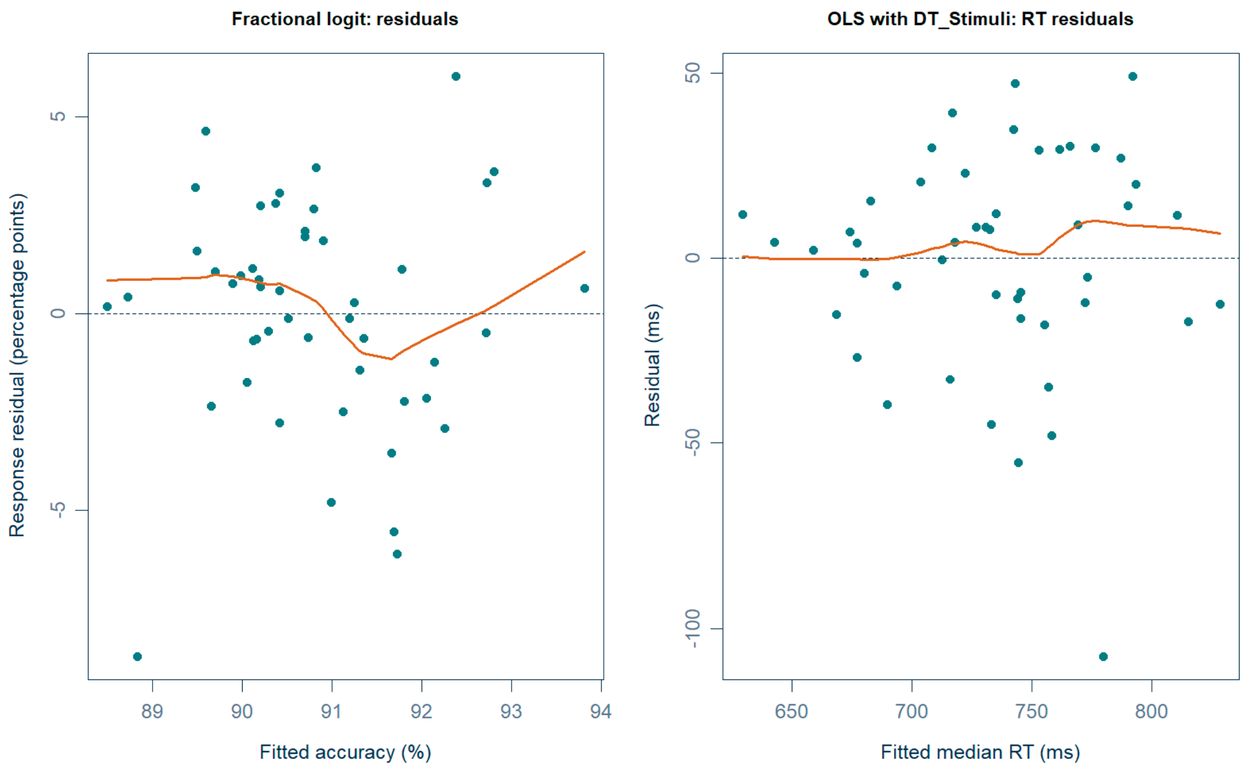

Figure S2. Residual displays for the accuracy fractional-logit model (left) and median-RT OLS model (right), each containing HR change, baseline HR, and DT_Stimuli. The smooth curves show descriptive residual patterns.
